# Engineering Strategies for Suppressing the Shuttle Effect in Lithium–Sulfur Batteries

**DOI:** 10.1007/s40820-023-01223-1

**Published:** 2023-11-10

**Authors:** Jiayi Li, Li Gao, Fengying Pan, Cheng Gong, Limeng Sun, Hong Gao, Jinqiang Zhang, Yufei Zhao, Guoxiu Wang, Hao Liu

**Affiliations:** 1 Joint International Laboratory On Environmental and Energy Frontier Materials, School of Environmental and Chemical Engineering, Shanghai University, Shanghai, People’s Republic of China; 2 Centre for Clean Energy Technology, University of Technology Sydney, Broadway, Sydney, NSW 2007 Australia

**Keywords:** Shuttle effect, Designed strategies, Li–S battery, Lithium polysulfides

## Abstract

Lithium–sulfur (Li–S) batteries are supposed to be one of the most potential next-generation batteries owing to their high theoretical capacity and low cost. Nevertheless, the shuttle effect of firm multi-step two-electron reaction between sulfur and lithium in liquid electrolyte makes the capacity much smaller than the theoretical value. Many methods were proposed for inhibiting the shuttle effect of polysulfide, improving corresponding redox kinetics and enhancing the integral performance of Li–S batteries. Here, we will comprehensively and systematically summarize the strategies for inhibiting the shuttle effect from all components of Li–S batteries. First, the electrochemical principles/mechanism and origin of the shuttle effect are described in detail. Moreover, the efficient strategies, including boosting the sulfur conversion rate of sulfur, confining sulfur or lithium polysulfides (LPS) within cathode host, confining LPS in the shield layer, and preventing LPS from contacting the anode, will be discussed to suppress the shuttle effect. Then, recent advances in inhibition of shuttle effect in cathode, electrolyte, separator, and anode with the aforementioned strategies have been summarized to direct the further design of efficient materials for Li–S batteries. Finally, we present prospects for inhibition of the LPS shuttle and potential development directions in Li–S batteries.

## Highlights

- The electrochemical principles/mechanism of Li–S batteries and origin of the shuttle effect have been discussed.
- The efficient strategies have been summarized to inhibit the shuttle effect.
- The recent advances of inhibition of shuttle effect in Li–S batteries for all components from anode to cathode.

## Graphical abstract

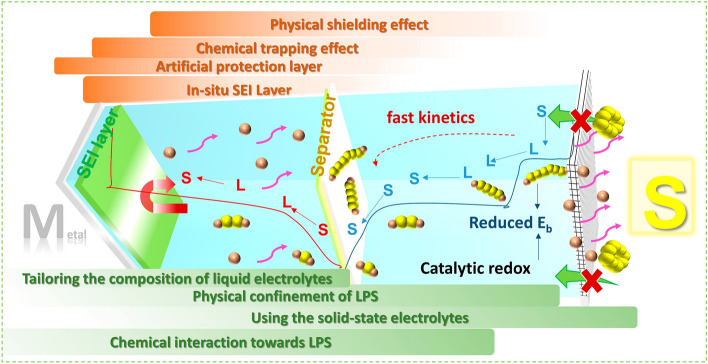

## Introduction

Rapidly depleting fossil fuel resources coupled with increasing environmental pollution has accelerated the pace of developing environmentally sustainable and high-energy–density renewable energy [1–3]. Lithium-ion batteries (LIBs), as a new clean energy source, have become important energy storage candidates in the electronic and communication equipment market [4]. However, their restricted energy density (150–240 Wh kg^−1^) and lack of memory retention render them unsuitable for deployment in grid and hybrid/electric vehicle. Recently, lithium–sulfur (Li–S) batteries, as rechargeable batteries incorporating multi-electron chemistry, have garnered intensive attention [5–7]. Their theoretical capacity (1675 mAh g^−1^) is much higher than that of LIBs (e.g., 274 mAh g^−1^ for lithium cobalt oxide (LiCoO_2_)), and even surpasses those of selenium and tellurium-based batteries (678 and 419 mAh g^−1^, respectively). Moreover, sulfur is abundant and environmentally friendly, making Li–S batteries competitive for widespread deployments [8]. Even though Li–S batteries possess appealing advantages, several challenges still limit their practicality: (i) the intrinsic electrical insulation (5 × 10^–30^ S cm^−1^) and volumetric expansion of sulfur; (ii) the reaction between the Li anode and the electrolyte resulting in unstable solid electrolyte interphase formation (SEI) and dendrite formation due to non-homogeneous nucleation at anode; (iii) the shuttling effect initiated by the polysulfides dissolution [9].

To solve the aforementioned problems, improvements have been made to different components of the battery [10–25]. Specifically, to solve the intrinsic insulating properties of sulfur, carbon materials (e.g., active carbon, carbon nanotubes (CNT) [26] (108 S m^−1^), graphene [27], and their composites [28–30]) have been designed and prepared to improve the overall conductivity of the electrodes [30–32]. Specifically, an impressive capacity of 1006 mAh g^−1^ at 2 C was observed for the super-aligned CNT/S [33]. To reduce lithium dendrites and undesired side reactions, in situ or artificial SEI membranes are a one-two-punch approach to both insulate the electrolyte and avoid polysulfide attack on the lithium metal [31]. These will motivate additional efforts including electrode structure, electrolyte solvent, and electrolyte additive screening to customize the composition/structural characteristics of the SEI layer and the associated ion transport at the interface. Another strategy for the anode is to substitute Li metal with Li-free anodes (e.g., Li_2_S) for a new Li–S battery configuration, which has been identified as one of prospective directions to achieve ideal anodes [33]. Naturally, Li anode is accommodated in the 3D current collector with a submicron skeleton [32] may well be an ideal candidate to take on this task [34].

Although the above-mentioned strategies can solve the first two issues, the shuttle effect in liquid electrolyte still significantly challenges the efficient sulfur reduction reaction (SRR) within Li–S batteries, which heavily damages the cathode, electrolyte, and lithium metal [35]. Soluble polysulfides diffuse toward the anode side, where they react with lithium metal to produce insoluble and insulating Li_2_S_2_/Li_2_S [36, 37]. This reaction leads to the loss of sulfur material and reduces Coulombic efficiency. So far, many strategies to curb the shuttle effect have been developed and effectively improved the utilization rate of sulfur in the cathode, and thus boosted the electrochemical performance of Li–S cells [38]. For instance, nonpolar carbon-based materials not only act as a conductive carrier of sulfur but also physically confine LPS [39, 40]. In recent times, a synergistic approach that involves a combination of multiple functional materials (e.g., heterogeneous atoms [41–43], catalysts with double defects [44–46], and heterostructure materials [47, 48]) has been devised, which possesses appropriate adsorption and catalytic capabilities to effectively execute SRR and enhances the performances of Li–S batteries [49]. The enhanced performances are ascribed to the promoted bidirectional conversion of LPS as a redox accelerator and regulated uniform Li plating/stripping to slow the growth of Li dendrites [50]. Moreover, tailoring electrolyte systems to construct an anode SEI layer [51] and a cathode solid electrolyte interphase (CEI) layer form on both electrodes by molecular regulation of electrolytes using optimal solvents/co-solvents [51–55], highly concentrated electrolytes (HCE) [56–61], and electrolyte additives [62, 63], etc*.* with various numbers of anchoring sites which significantly improved the stability of the Li anode interface, controlled the kinetics of sulfur redox, and suppressed side reactions toward polysulfides. Consequently, there is an improvement in the retention rate of capacity.

**Fig. 1:**
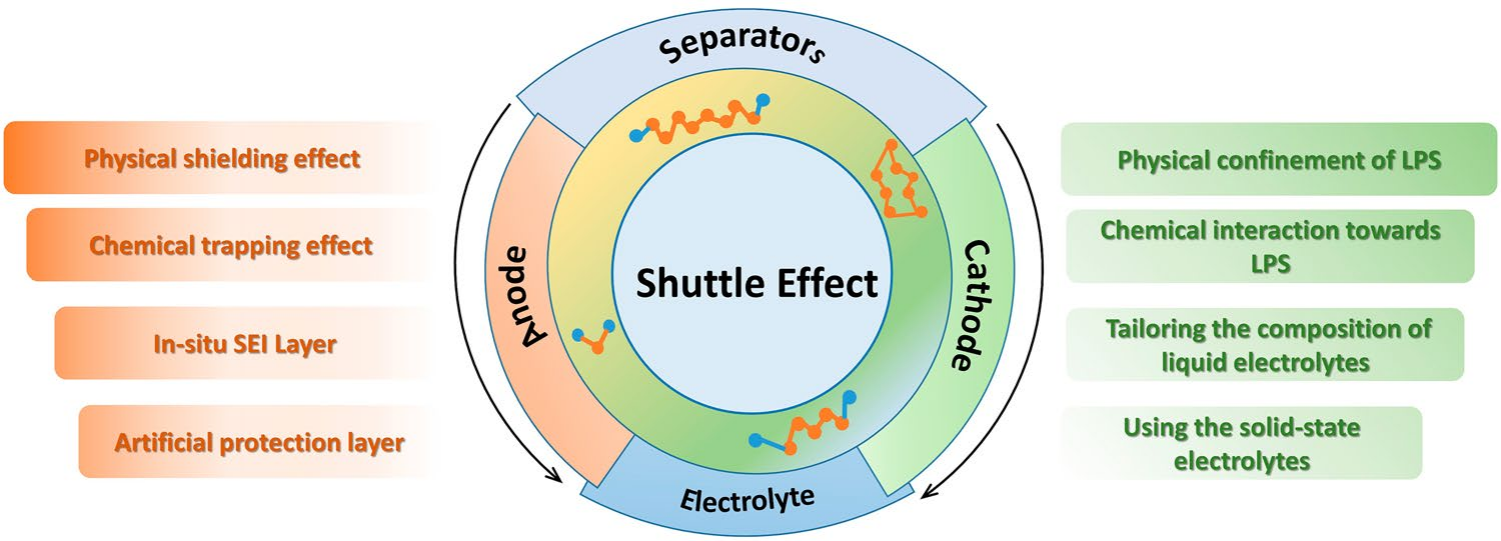
Schematic illustrations of the strategies and operation mechanisms for inhibiting LPS shuttling starting

Extensive research has gone into the development of efficient strategies to inhibit the shuttling effect and achieve excellent performance in Li–S batteries [64–66]. Several reviews have summarized the design of cathode materials, high-sulfur loading, the inhibition of shuttle effect on the anode or electrolyte [67], etc*.* However, a comprehensive and systematical review regarding the strategies for suppressing the shuttling effect for all components of Li–S batteries is lacking and desired, especially for their practical application in future commercialization. In this review, we center on the shuttle effect issues and suppressing strategies in Li–S batteries (Fig. 1). We will first discuss the electrochemical principles and shuttle effect of Li–S batteries to give an overview of the mechanism and original of the shuttle effect. The designed principles for prohibiting LPS shuttle will be elaborated, including boosting the sulfur conversion rate of sulfur, confining sulfur or LPS within cathode host, confining LPS in the shield layer, and preventing LPS from contacting the anode, which offers guidance for further design novel materials of Li–S batteries. Then, we summarize the inhibition of shuttle effect from all components in Li–S batteries (cathode, electrolyte, separator and anode) with the above-designed principles. Finally, the prospects for inhibition of the shuttle effect and future development directions in Li–S batteries will be elucidated.

## Electrochemical Principles and Shuttle Effect of Li–S Batteries

A typical half Li–S cell comprises sulfur cathode, separator, electrolyte, and lithium metal anode. And a conversion-type working mechanism is inherited during charge/discharge process. Specifically, each sulfur atom undergoes a complete two-electron redox reaction:

1 $${\text{S}}_{8} \; + \;16{\text{Li}}^{ + } \; + \;16e^{ - } \; \leftrightarrow \;8{\text{ Li}}_{2} \;{\text{S}}$$

**Fig. 2:**
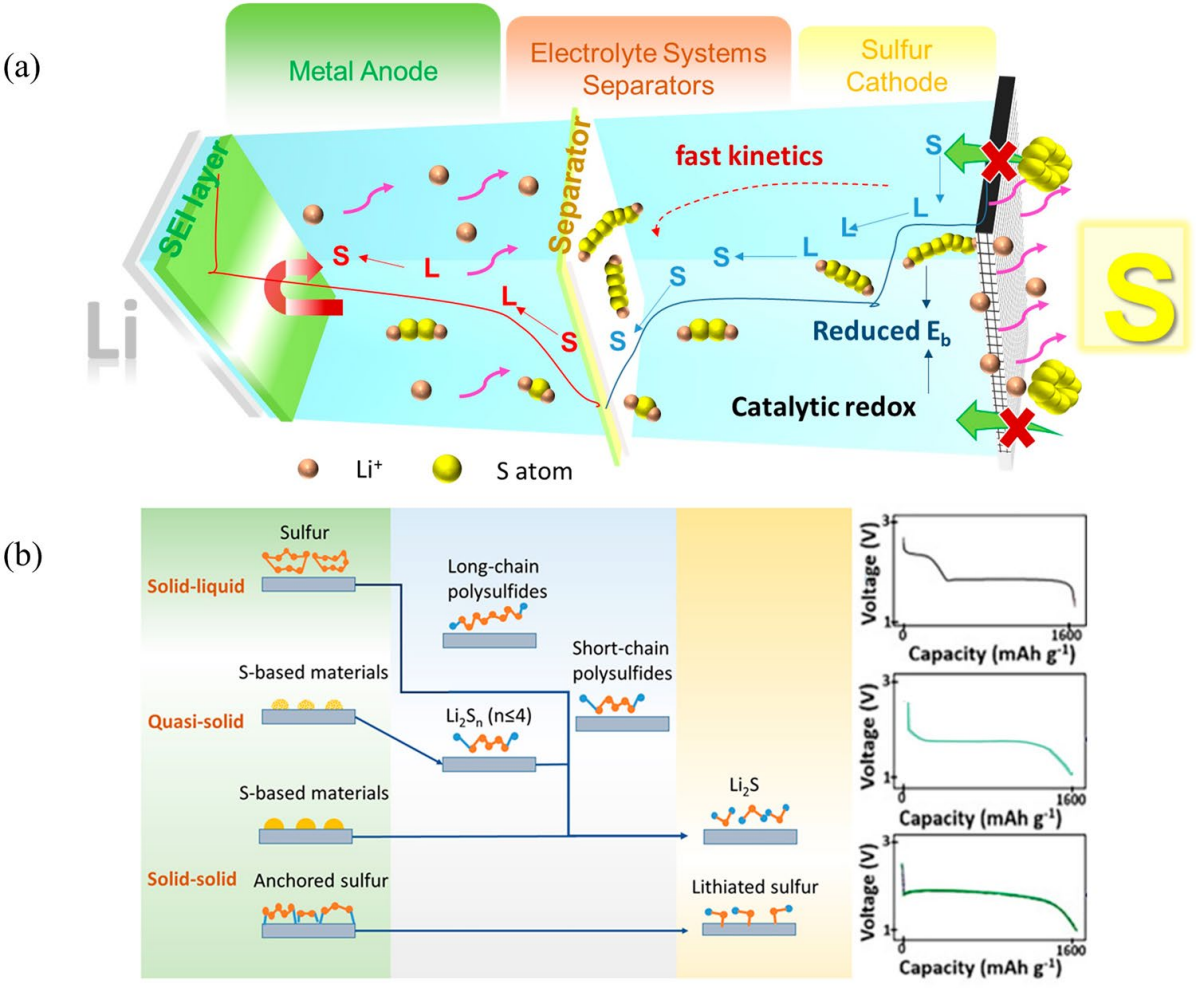
**a** Schematic diagram of Li–S battery containing differnt components. **b** Response mechanisms of “solid–solid,” “quasi-solid,” “solid–liquid-solid,” and corresponding discharge profiles

As shown in Fig. 2a, Li–S batteries typically show two plateaus during discharging, wherein S electrochemically reduces to Li_2_S via soluble intermediate polysulfides, i.e., S_*k*_^−2^ (4 ≤ *k* ≤ 8), relating to a “solid–liquid-solid” process. Specifically, the discharge voltage plateaus at ~ 2.35 V contribute to 25% of the total theoretical specific capacity (419 mAh g^−1^). The electrochemical reduction of this part goes through two stages. Initially, solid S_8_ transforms into soluble higher-order Li_2_S_8_ upon reaction with migrating Li-ions and electrons, relating to the reaction of converting between solid–liquid [68]. Subsequently, the highly soluble higher-order Li_2_S_8_ tends to be disproportionate in the aprotic electrolytes and lower-order polysulfide anions (S_*k*_^−2^, *k* > 4) produced by single-phase liquid–liquid reactions. The low discharge voltage plateaus (< 2.1 V) represent a further reduction of these lower-order intermediate polysulfides to solid state products (Li_2_S_2_/Li_2_S), which contributes to the 75% of the total specific capacity (~ 1256 mAh g^−1^). This part undergoes a two-stage electrochemical reduction as well. In the first stage, soluble lower-order polysulfides are reduced to insoluble Li_2_S_2_ or Li_2_S, and this process is a slow two-phase reaction involving liquid–solid phases [69]. In the last ramp stage, Li_2_S_2_ dissociates to Li_2_S eventually, two low-conductivity solid-phase transformation processes that is always subject to large polarization and slow kinetics. Reversible solid–liquid-solid reactions also occur in the oxidation process (charging) whereby the Li_2_S is converted to elemental S through dissolved intermediate polysulfides [70].

The reaction about sulfur in Li–S batteries often leads to an internal shuttle effect, particularly during the “solid–liquid-solid” process. This issue can be solved by transitioning to quasi-solid or solid–solid transformation, which will be explained in Sect. 3. The soluble intermediate polysulfides move toward the anode driven by the concentration gradient and then combine with the Li anode to form lower-order polysulfides. As short-chain polysulfides experience greater electric field force than concentration gradient force, they move back toward the cathode and cause the production of higher-order polysulfide. The ongoing process of shuttle effect may result in significant self-discharge of the anode due to corrosion, and poor Coulombic efficiency (CE).

In particular, the soluble polysulfides would become disconnected from the current collector, which would separate them from engaging in future electrochemical reactions. This would result in significant sulfur loss, leading to a subsequent decrease in the CE. Furthermore, the non-dissolvable layer of Li_2_S_2_/Li_2_S that collects on the Li anode surface would not turn into long-chain LPS and S_8_ again due to their deficiency in electrical conductivity. As a result, the active materials will be permanently lost, and the diffusion and transfer of Li^+^ ions will be delayed, resulting in a rapid decline in capacity and a short cycle life. Moreover, when the electrolyte viscosity increases due to the dissolution of LPS, the resistance to charge transfer in Li–S batteries also increases. This occurs because the solid Li_2_S_2_/Li_2_S during oxidation encounters a nucleation barrier, and Li_2_S_2_/Li_2_S decomposes and requires extra activation energy during discharge. Moreover, when the electrolyte viscosity increases resulting from the dissolution of LPS, the resistance to charge transfer also increases in Li–S batteries. The observed phenomenon may result from the nucleation barrier encountered during the oxidation of solid Li_2_S_2_/Li_2_S, as well as from the decomposition of Li_2_S_2_/Li_2_S which requires overcoming additional activation energy during reduction.

## Design Principles for Prohibiting Shuttle Effect of Li–S Batteries

A complex reaction kinetics, multi-step phase transition, accompanied by the shuttle effect, is generally involved in the operation of Li–S batteries. Shuttle effect represents a significant challenge for achieving efficient SRR within Li–S batteries. The phenomenon can cause considerable damage to all battery components. At the sulfur cathode, sulfur has a significant reaction barrier with Li-ions during the electrochemical cycle, as shown by the overpotential. This leads to sluggish reaction kinetics, prolonged retention time of polysulfide intermediates, an exacerbated shuttle effect, as well as a reduction in both the electrochemical stability and lifetime of the cell. This means that Li–S batteries can achieve stable cycling and high energy density if the shuttle effect is effectively suppressed. In this section, we will discuss the design strategies to prevent the shuttle effect through boosting the sulfur conversion rate, confining sulfur or LPS within cathode host, confining LPS in the shield layer, and preventing LPS from contacting the anode.

### Boosting the Sulfur Conversion Rate

The shuttle effect is inevitable in the traditional solid–liquid-solid conversion process, but if the conversion process of the sulfur-lithium reaction process can be changed to avoid the formation of soluble polysulfide or reduce the existence time of polysulfide, this will be a fundamental way for eliminating the shuttle effect. Researchers have developed two alternative conversion mechanisms for Li–S batteries. One is a quasi-solid conversion mechanism whereby the activated sulfur can be directly reduced into the short-chain substance Li_2_S_*x*_ (where x is less than or equal to 4); this substance is then reduced to form solid Li_2_S [71]. Through this reaction pathway, the formation of higher-order LPS intermediates is minimized; in turn, this prevents the detrimental parasitic shuttling that afflicts Li–S batteries [72]. A typical quasi-solid conversion pathway behaves a single-platform discharge process, with a capacity-voltage curve that is usually characterized by a weakened high plateau (≈2.3 V) and an extended discharge plateau (< 2.1 V) in first cycle (Fig. 2b). The other is solid–solid pathway which only involves the conversion between S and Li_2_S, avoiding the production of intermediates. So, it is always a single slope platform curve at 1.70 V [73]. Therefore, achieving the quasi-solid/solid–solid transition mechanism is main approach to boosting the sulfur conversion rate.

#### Ultra-microporous Carbon Confined Small Molecules

**Fig. 3:**
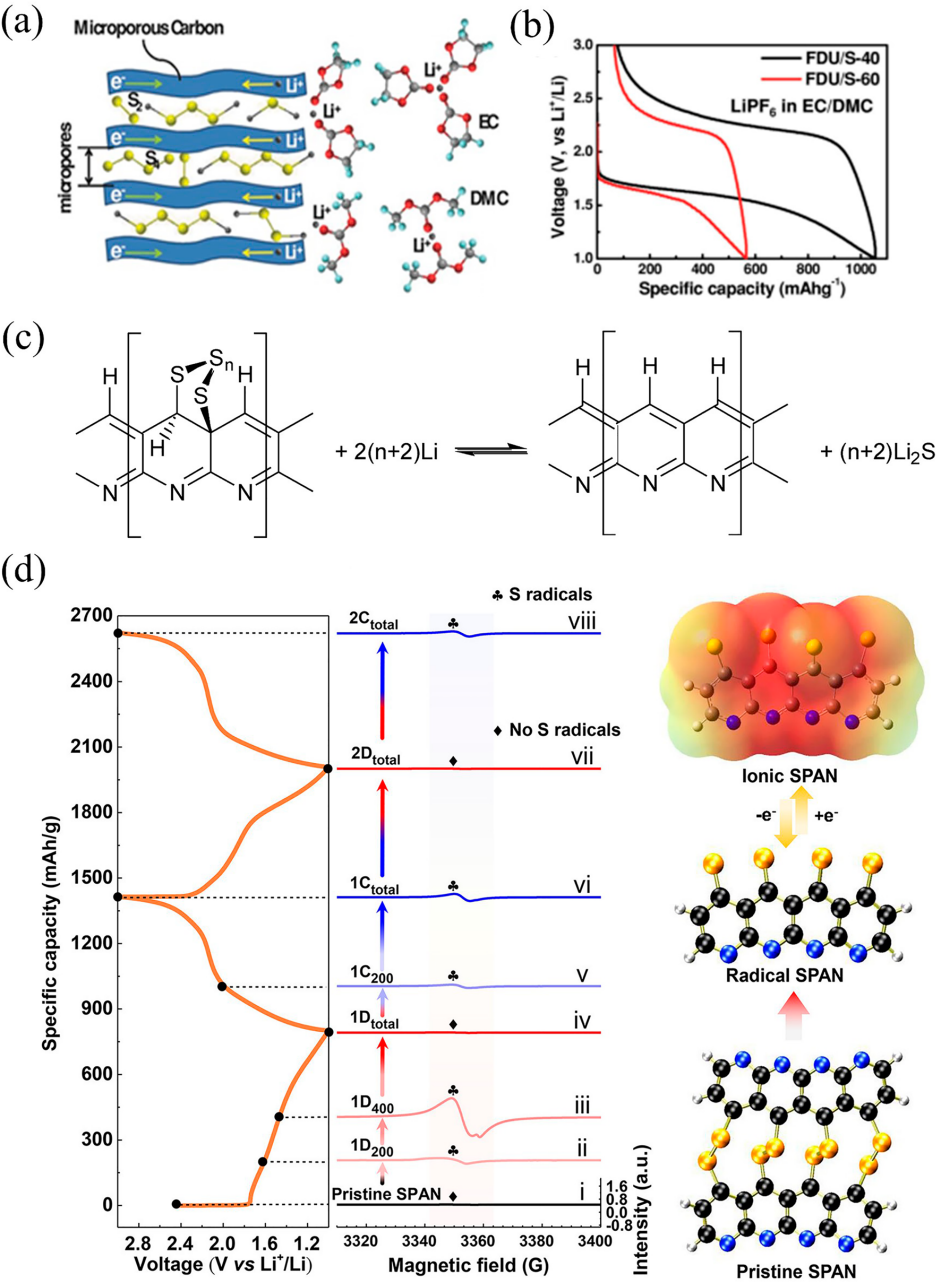
**a** Schematics of the lithiation process for UMC/S and mesoporous carbon/S cathode in carbonate-based electrolyte. **b** Electrochemical curves of FDU/S-40 and FDU/S-60 [74]. Copyright 2014, Wiley–VCH. **c** Overall reaction of Li/SPAN cell [78]. Copyright 2014, MDPI. **d** The lithiation process of SPAN from ex situ electron paramagnetic resonance (EPR) spectra [79]. Copyright 2018, American Chemical Society

A practical way of reducing the dissolution of active material and thus the shuttle effect, is to confine sulfur with ultra-microporous carbon (UMC) whose pore size ≤ 0.7 nm. The sulfur confined in such micropore structures can be existed as small molecules due to steric constraints, such as S_2−4_ [74, 75]. At the same time, UMC materials prevent solvent molecules from entering the pores because they have a smaller size than the solvated ion (with EC and DMC having calculated diameters of 5.74 and 7.96 Å, respectively) (Fig. 3a) [76]. On the other hand, the solvent molecules become highly distorted, which would lead to the solvation energy turns lower than surface energy of the sub-nanopore, which forces the Li^+^ to desolvate and then moves through the carbon to react with sulfur [74]. Therefore, regardless of the electrolyte in use (whether ether or carbonate based), the concentration of solvent molecules inside the pore is almost negligible, resulting in a quasi-solid reaction mechanism [77]. For instance, a highly ordered microporous carbon FDU (0.46 nm) is used as the confinement matrix for S_2−4_ composites (FDU/S-40) in different electrolytes, exhibiting comparable electrochemical behavior: single discharge plateau at approximately 1.8 V, initial reversible capacity above 1000 mAh g^−1^, and stable cycling (Fig. 3b).

#### Sulfur-conjugated Organic Skeleton (Organic Sulfides/Sulfur-containing Polymers)

Sulfur-containing polymers have exhibited positive attributes in Li–S batteries with solid–solid conversion owing to chemically bonded short-chain sulfur, in which element S existed as short -S_2_- and -S_3_- chains through reversible C–S/S–S bonds and transformed exclusively to Li_2_S to facilitate the solid-to-solid process. Li–S batteries achieve superior cycling stability compared to conventional cyclo-S_8_ cathodes, which generate lithium polysulfide (LPS) during cycling, due to the fundamental elimination of the shuttle effect [80]. Specifically, covalent attachment of S_*n*_ (*n* = 2–4) species to the PAN backbone was achieved through a one-step pyrolysis of sulfur and commercial polyacrylonitrile (PAN). Figure 3c displays the formula of lithiated SPAN, where sulfur was covalently bonded to the π-conjugated carbon skeleton through C–S bonds [78]. Furthermore, SPAN is optimally matched with the carbonate electrolyte, a typical SPAN discharge/charge curve (Fig. 3d) shows that no LPS is generated. During the first discharge, sulfhydryl radicals are produced as the S–S bond in the pristine SPAN is broken, as shown in Fig. 3d. Encapsulating these smaller sulfur molecules in the cathode, while covalently bonding and physically constraining, completely eliminates LPS dissolution and shuttling between the anode and cathode [79].

Moreover, porous organic polymers with much larger specific surface areas and more accessible pores can efficiently eliminate the volume expansion of sulfur cathode and enable higher sulfur loading (> 50%). An illustration of this can be with the preparation of the graphdiyne (GDY) type of the porous organic framework (GPOF). The pyrene nodes' π–π interaction resulted in interconnected channels in planes, providing an accommodation for more sulfur species in GPOF. Moreover, the acetylenic groups enriched with electrons are highly reactive, facilitating the combination with sulfur molecules in the form of C–S–S–C in the nanochannel and by solid-phase conversion to inhibiting the shuttle effect [81]. Therefore, GPOF-S composite enabled 56.8 wt% sulfur loading and average discharge capacities of 925 mA h^−1^ at 0.2 C, accompanied by a negligible reduction in capacity after 250 cycles.

#### Sulfur-rich Compounds/Sulfur-Containing Inorganic Compounds

**Fig. 4:**
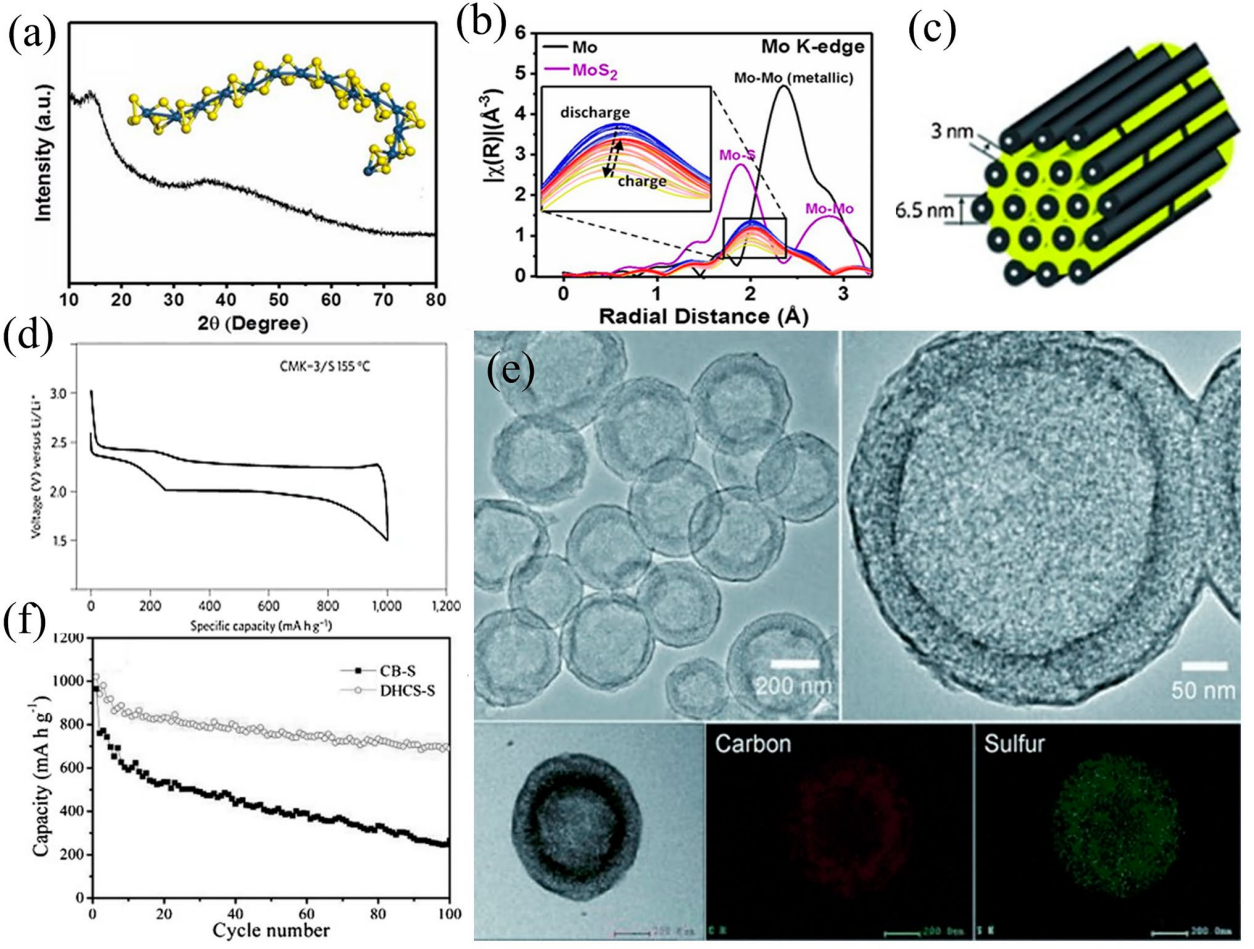
**a** XRD pattern and **b** Fourier-transformed Mo K-edge EXAFS spectrum of 1D chain-like MoS_3_ [82]. Copyright 2017, Natl Acad Sciences. **c** A schematic diagram of the sulfur (yellow) confined in CMK-3. And **d** galvanostatic electrochemical curves of the first cycles with CMK-3/S [84]. Copyright 2009, Nature Portfolio. **e** TEM images and elemental mapping of DHCS-S. **f** Electrochemical performance of DHCS-S and carbon black-sulfur (CB-S) [85]. Copyright 2012, Wiley–VCH. (Color figure online)

Instead of directly using elemental sulfur, the sulfur-containing compounds with incorporating the transition metals (e.g., Mo, Fe, Ti, Nb) have exhibited sulfur-like electrochemical behaviors [82]. Sulfur-rich compounds operate on a single voltage profile platform through the reversible breaking and formation of S–S bonds, lithium-ion insertion and extraction mechanisms. For instance, MoS_3_ has been used as cathode material replacing the pure element sulfur, which has a chain-like structure composed of Mo ions bridged by sulfide and disulfide ligands (as shown in Fig. 4a), enabling highly efficient active storage and rapid ion transfer [83]. Additionally, the amorphous chain-like structure of MoS_3_ is mostly maintained during lithiation/delithiation process with no Mo–S bond broken, and also no LPS intermediates generation has been observed in the electrochemical reaction process revealed by operando X-ray absorption spectroscopy (Fig. 4b).

Quasi-solid and solid–solid reactions are effective in addressing major challenges of Li–S batteries e.g., the shuttle effect caused by polysulfides and high dependency on electrolyte consumption. Nonetheless, the practical energy density of Li–S cells is significantly restricted due to the low sulfur content and inert redox kinetics of such cathodes [86].

### Confining Sulfur or LPS within Cathode Host

To increase the performance of Li–S batteries with solid–liquid-solid conversion, the strategies focus on inhibiting polysulfide dissolution or catalyzing the rapid conversion of sulfur into low-solubility discharge products [5, 33, 38, 87]. At the same time, so as to achieve the practical energy density, the electrolyte should be minimized, e.g., lean electrolyte with the electrolyte/sulfur ratio (E/S ratio) < 10 μL mg^−1^. However, most of the achievements in the last decade have been based on the excessive use of electrolytes. In this section, physical confinement, chemical anchoring and electrochemical catalysts strategies to inhibit the shuttle effect will be presented by designing optimized sulfur hosts that provide a high conductivity/ionic conductivity environment and fast redox kinetics. The recent host materials are summarized in Table 1 [88–102].

**Table 1:** Summary of recent results on “Confining sulfur or LPS within cathode host”

Cathode host	Sulfur loading (mg cm^−2^)	Capacity (mAh g^−1^)/C rate/cycles	Fading rate (%)	S loading (wt%)	E/S (μL mg^−1^)	Refs.
3DIO FCSe–QDs@NC	1.5–2	801/1/500	0.035	70.2	20	[88]
S–TVTCSi_4_–KJB_2%_	1	652/0.5/500	0.037	80.8	25	[89]
S@CNTS/Ni–PC	2	408/2/500	0.1	30	25	[90]
Co–NCNT@HC	1.2	750/1.2/1000	0.037	73.5	–	[91]
sGNC–S	2–3	829.3/0.5/200	0.155	74.3	–	[92]
Nb_3_VS_6_	1.5	759/2/1000	0.017	–	–	[93]
S/Ni–Co	3.84	~ 600/1/200	0.082	74.3	8	[94]
NiO–MoS_2_@CNFs	1.5	973.3/1/500	0.018	–	12	[95]
v-ZnTe/CoTe_2_@NC/S	1.6	742/1/500	0.022	68	20	[96]
Vanadium-doped MoS_2_ (VMS)	7.2	725/1/300	0.06	75	14.7	[97]
TiO_2_–B	2	572/0.2/100	0.34	61	10	[98]
TiO_2_/BaTiO_3_	1.2	541/0.5/500	0.08	58	10	[99]
HMCS@GO	1	626/0.2/300	0.16	58.9	–	[100]
SnS_2_@NHCS	1	395/1/500	0.065	69.7	–	[101]
Fe_3_C/N–CNF@RGO	1.6	781/0.5/300	0.009	62.5	15	[102]

#### Physical Confinement Method for Sulfur or LPS

Considerable effort has gone into solving the shuttle effect. The materials have been designed with a complex internal path and porous, absorbent carbon, to physically confine the sulfur or LPS in the cathode side [103], which strongly inhibit the bulky polysulfide anions from diffusing out of the channels into the electrolyte. Nazar and colleagues conducted a pioneering work that employed mesoporous carbon (CMK-3) as a conductive host material to trap or encapsulate S/Li_2_S-active material and LPS (Fig. 4c) [84]. The resulting composite showed a capacity of 1005 mAh g^−1^, which was superiority over that reported in the literature for C-S composites (averaging between 300 and 420 mAh g^−1^) [104] (Fig. 4d). Subsequently, the preparation of sulfur hybrids with macro/meso/microporous carbons [105–107] and carbon nanofibers [108, 109], spheres [110], nanotubes (CNTs) [108] was reported using a similar physical confinement method with some of the advantages of large surface area and short Li^+^ ions diffusion paths [39]. One example is double-shelled hollow carbon spheres (DHCS) with intricate shell architectures, which can further enhance the benefits of hollow nanostructures [111, 112], enabling high levels of sulfur encapsulation, limiting outward diffusion of LPS, and withstanding volume changes during long-term cycling (Fig. 4e). The distribution of carbon and sulfur is mainly concentrated in the area between the two carbon shells. The analogous distribution of these two elements indicates that sulfur has a strong attraction to carbon. In Fig. 4f, a better capacity retention was achieved using DHCS-S as the cathode (initial discharge capacity was 1020 mAh g^−1^, second cycle remained at 935 mAh g^−1^) [85].

#### Chemical Anchoring Techniques for Effective Sulfur or LPS Confinement

**Fig. 5:**
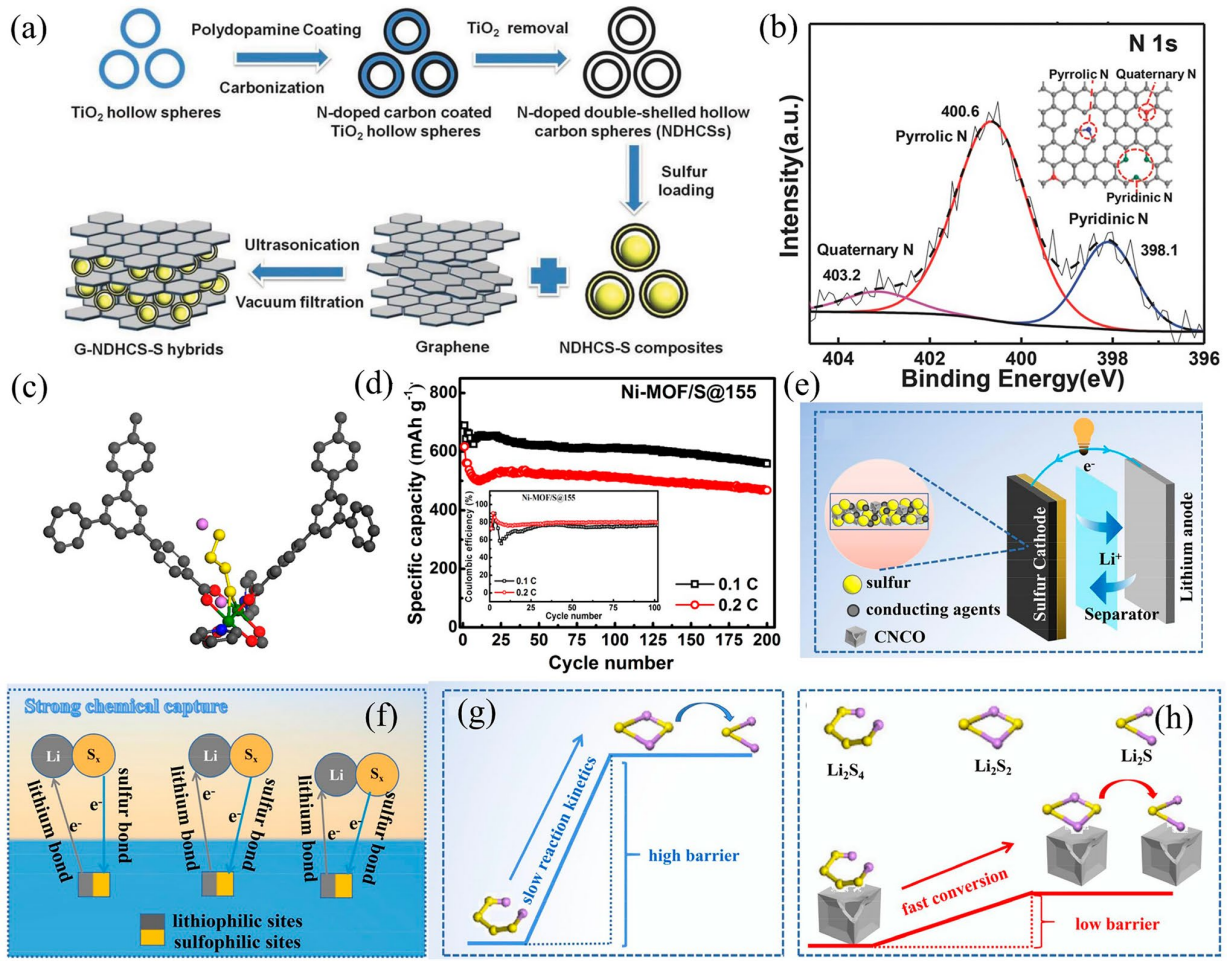
**a** Preparation process and **b** N 1*s* XPS spectra of the G–NDHCS–S [113]. Copyright 2015, Wiley–VCH. **c** Schematic diagram of the interaction between LPS and Ni–MOF. **d** Electrochemical performance of Ni-MOF/S composite [116]. Copyright 2014, American Chemical Society. **e** Schematic diagrams, **f** lithiophilic/sulfiphilic dual binding sites, and **g**, **h** SRR catalyst of CNCO as additive in Li–S battery [117]. Copyright 2022, Elsevier

While the carbon–sulfur composites exhibit exceptional electrochemical behavior during the initial charge/discharge cycle, a pronounced degradation has been observed in subsequent cycles. This decline can account for the fact that mere physical confinement does not sufficiently expedite the kinetic processes involved in LPS transition. Additionally, the composites lack the requisite adsorption capacity to effectively mitigate LPS dissolution. Consequently, chemical anchorage strategies have been implemented to enhance inhibition of polysulfide solvation, including heteroatom-doped carbons, conjugated polymers, transition metal oxides, nitrides, and sulfides. Heteroatom-doping (N, O, S, P, Se, etc*.*) and polar materials have been studied for their ability to entrap the soluble LPS effectively was accounted for polar-polar interactions. For example, graphene wrapping nitrogen-doped double-shelled hollow carbon spheres (G-NDHCS-S) have been designed (Fig. 5a). The introduction of nitrogen atoms creates reactive sites for rapid charge transfer, allowing the hollow carbon spheres to immobilize more polysulfide ions (Fig. 5b), delivering a high initial discharge capacity of 1360 mAh g^−1^ at 0.2 C and rate performance of 600 mAh g^−1^ at 2 C [113]. Another way is to form coordinate bonds based on Lewis acid–base interactions, in which polysulfide anions (S_*x*_^2−^, 4 ≤ *x* ≤ 8) as a Lewis base and most representative Lewis acid from metal ions of metal–organic frameworks (MOFs) [114] or MXenes [115]. As shown in Fig. 5c, soluble polysulfide ions are trapped in the MOF scaffold by the Lewis acidic Ni(II) center of the Ni-MOF, as evidenced by the capacity retention of up to 89% after 100 cycles at 0.1 C [116] (Fig. 5d).

The inclusion of catalysts is necessary to improve the sluggish conversion kinetics of polysulfides as a means to reduce the excessive accumulation of long-chain LPS and improve the electrochemical performance. Specifically, with the addition of an electrocatalyst to the sulfur electrode, the conversion process of S will only occur in cathodes, which means that the “solid–liquid-solid” transform of LPS takes place inside the cathode material. The transfer of lithium ions is realized by wetting the electrode with electrolyte [118–120]. Thus, as shown in Fig. 5e-h, hollow Co_*x*_Ni_1-*x*_O concave (CNCO) as an additive endowed cathode host with strong affinity and efficient SRR catalysts which derived from sites with dual binding affinity to lithophile and chalcophile elements and abundant oxygen vacancies of CNCO. The CNCO/S can provide a high initial specific capacity of 1355 mAh g^−1^ at 0.1 C with a low nucleation barrier and overpotential of Li_2_S [117] and under lean electrolyte (E/S = 9 μL mg^−1^) keep 796 mAh g^−1^ at 0.5 C.

**Fig. 6:**
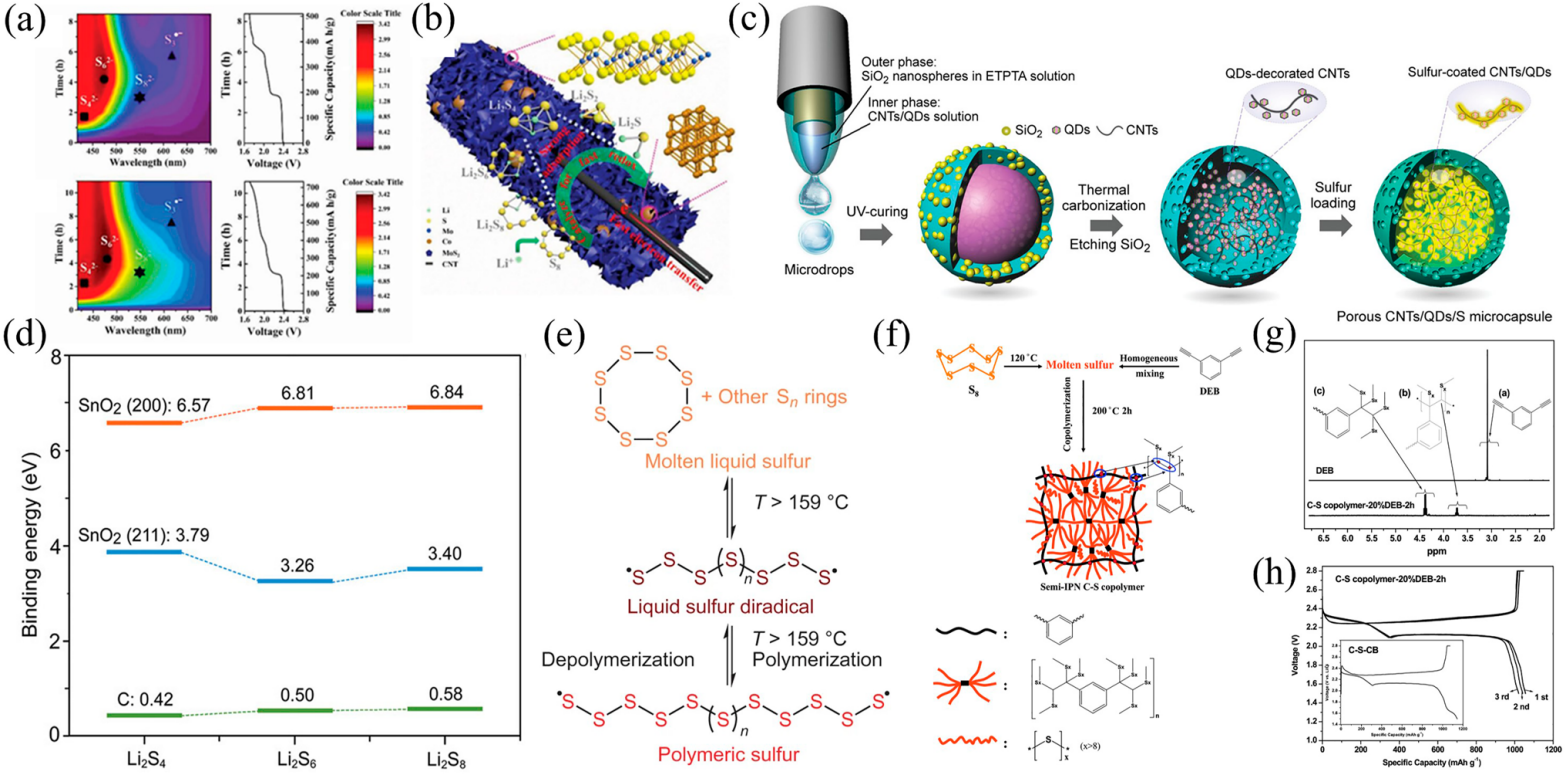
**a** In situ UV–VIS spectra of N–C and CNT/MoS_2_–Co composite S host; **b** Illustrates the interaction between CNT/MoS_2_–Co and LPS [124]. Copyright 2021, Wiley–VCH. **c** Preparation process of the porous CNTs/SnO_2_ QDs/S microcapsules; **d** Binding energies of polysulfides adsorbed on SnO_2_ and carbon [125]. Copyright 2021, Wiley–VCH. **e** Diagram of thermal ring-opening of S_8_ to polymeric sulfur diradicals [122]. Copyright 2013, Nature. **f** Fabrication of C–S copolymer fabrication; **g** ^1^H–NMR spectra and **h** initial three charge–discharge profiles of C–S copolymer composite [123]. Copyright 2014, The Royal Society of Chemistry

The direct usage of elementary sulfur when the formation of chemically stable copolymers is another method of chemical containment [121]. The dissolution and diffusion of LPS species from the cathode region are proficiently mitigated through robust chemical interactions established between the carbonaceous framework and sulfur constituents inherent in the C–S copolymers. However, their cycling performance is impeded by their inherent limitation in facilitating efficient electronic conduction. As shown in Fig. 6e, sulfur reacts with the polymer via organic radicals to produce a sulfur-rich polymer with a sulfur content of 90 wt% [122]. The resulting cathode exhibits an initial discharge capacity of 1100 mAh g^−1^ at 0.1 C, but capacity rapidly decreases to below 400 mAh g^−1^ at 2 C. Similarly, higher initial capacity (1143 mAh g^−1^ at 0.1 C) with inferior rate performance (595 mAh g^−1^ at 1 C) is observed for sulfur-rich polymer materials synthesized by copolymerization of elemental sulfur with 1,3-diethynylbenzene (as shown in Fig. 6f-h) [123].

#### Physical Confinement and Chemical Anchoring Co-existence for Effective Sulfur or LPS Confinement

The integration of physical confinement with chemical anchoring/catalysts as a sulfur host is a promising approach. An extraordinary nanostructure consisting of tube-in-tube carbon nanotubes (CNTs), sulfur-deficient molybdenum disulfide (MoS_2_) embedded with cobalt atom clusters has been developed as an effective regulator of LPS in Li–S batteries (Fig. 6b) [124]. Figure 6a illustrates a unique design that integrates physical confinement, chemical adsorption, and the kinetics of catalytic polysulfide redox reactions in a single package. In a similar manner, as shown in Fig. 6c, a cathode made of polysulfide-confined, porous microcapsules integrated with a composite core of carbon nanotubes, titanium dioxide quantum dots, and sulfur (CNTs/QDs/S) achieved a long life of 700 cycles, a high-sulfur loading of 2.03 mg cm^−2^, and a CE value of up to 99.9% [125]. The observed results were owing to the efficient adsorption of polar SnO_2_ quantum dots to LPS, which inhibited the shuttle effect. Moreover, the CNTs provided a fast electron transfer pathway, while the porous shell improved sulfur loading and electrolyte permeability. Additionally, the internal voids successfully adapted to the volumetric change of the sulfur during charging and discharging. (Fig. 6d.)

Sandwich structures serve as host materials, allowing both physical confinement and chemical anchoring by placing sulfur or its composites between two functional films, typically decorated with catalytically active materials. Functionalized films located at the edges of the sandwich structure enable physical confinement and catalytic conversion of the sulfur and its end-products within the sandwich. The film serves as a three-dimensional catalytic current collector and multifunctional interlayers. For example, by sandwiching sulfur and acetylene black (AB) (S/AB) between two 1 T–MoSe_2_/CC films (1 T–MoSe_2_/CC@S/AB@1 T–MoSe_2_/CC), the excellent catalytic activity and metallic properties of 1 T-MoSe_2_ can be used to lower the Gibbs free energy barriers for polysulfide conversion. While a single 1 T–MoSe_2_/CC membrane also inhibits the shuttle effect to some extent, sandwich structure is more suitable for the realization of high-sulfur Li–S batteries with a lean electrolyte. Therefore, the Li–S batteries with 1 T–MoSe_2_/CC@S/AB@1 T–MoSe_2_/CC allow a high capacity of 5.43 mAh cm^−2^ and cycle up to 200 times, which was suitable for lean electrolyte (E/S was 7.8 μL mg^−1^) and high-sulfur loading (5.7 mg cm^−2^) applications [126]. Using these strategies described above, sluggish kinetics and the shuttle effect can be overcome even under lean electrolyte conditions.

A lower E/S is necessary to utilize the high-energy-density potential of Li–S batteries. Nevertheless, the commonly employed sulfur cathode is typically solid–liquid-solid lithiation process, and to achieve full discharge capacity, a large amount of electrolyte is typically required to completely dissolve the long-chain LPS. In contrast, the solid–solid or quasi-solid-phase conversion pathway eliminates the long-chain LPS generation and significantly reduces the battery's dependence on electrolyte consumption. As a result, sulfur cathodes are more likely to achieve low E/S ratios through solid–solid conversion reactions.

### Confining LPS in the Shield Layer

The dissolution of LPS is inevitable in the liquid electrolyte. If the strategies for regulating the cathode are unable to fully avoid dissolving and spreading of LPS, It would spread to the interface of the separator, driven by the concentration gradient. This area between the cathode and the separator is called the shield layer. In this area, engineering cathode electrolyte interphase and constructing functional separators to prevent the shuttle of polysulfide are two potential methods.

#### Interfacial Protection and Engineering Peculiar Cathode Electrolyte Interphase

**Fig. 7:**
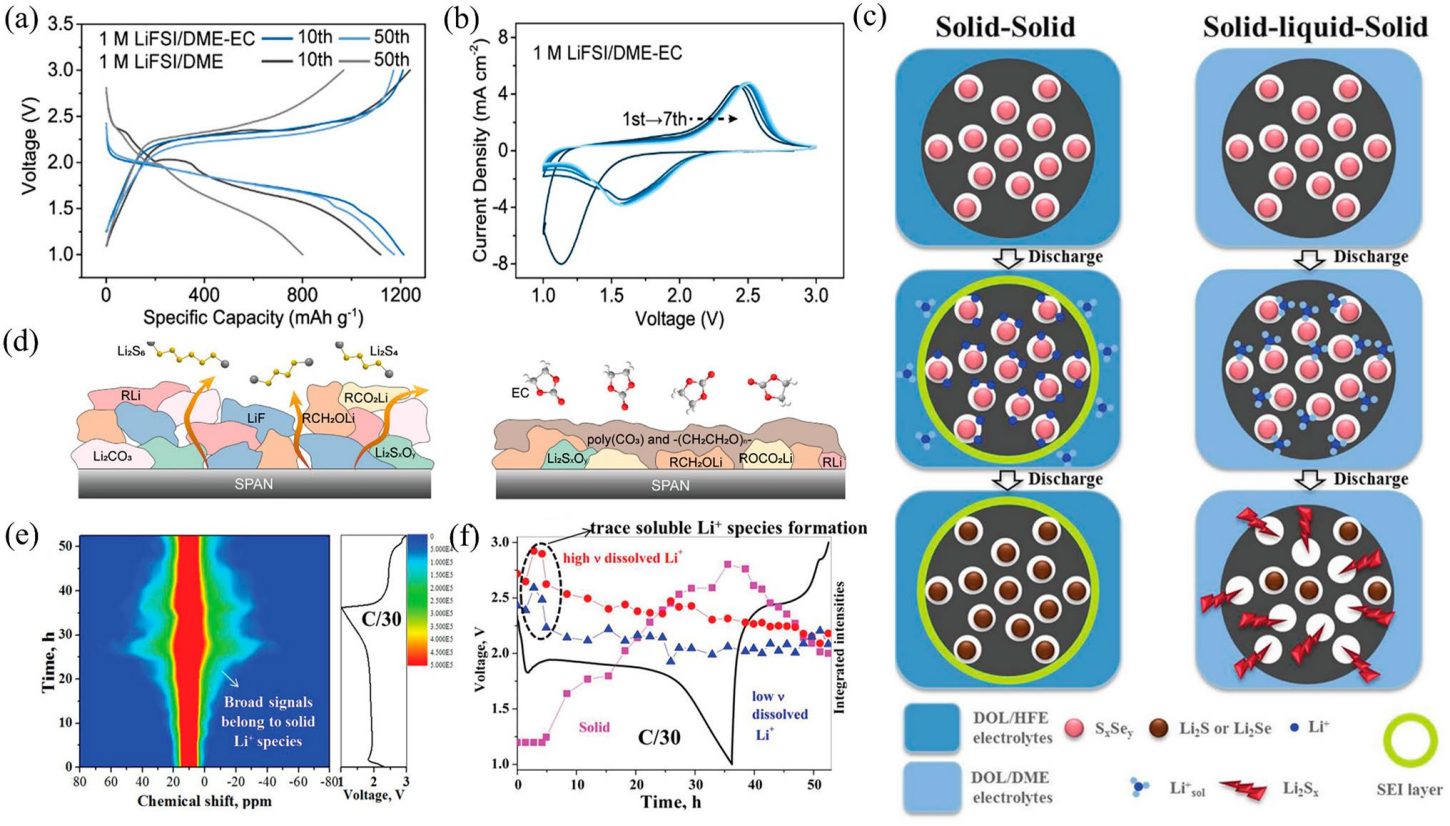
**a** Discharge–charge curves and **b** cyclic voltammograms (CV) of Li–SPAN cells [129]. Copyright 2021, American Chemical Society. **c** Different lithiation mechanism of Se–S cathodes in different electrolytes [131]. Copyright 2019, Wiley–VCH. **d** Schematic of the structure of CEIs in 1 M LiFSI/DME and 1 M LiFSI/DME-EC [129]. Copyright 2021, American Chemical Society. **e** Contour plot of NMR signal, and **f** Areas function of Li^+^ species consistent with the predicted voltage profile [131]. Copyright 2019, Wiley–VCH

In Li–S batteries, during the first few cycles of electrolyte decomposition, both an SEI and a CEI layers form on the electrodes [127]. Ideally, the SEI and CEI layers should fully develop after a few cycles and become passive, resulting in the formation of thin films created from the by-products of the partially reacted electrolyte solvent and decomposed salt fragments [128]. SPAN cathodes can run for more than 500 cycles in a carbonate-based electrolyte, whereas in an ether electrolyte polysulfide dissolution occurs, leading to rapid capacity degradation (70% capacity loss in 100 cycles) (see Fig. 7a, b). Therefore, it is logical to speculate that the CEI (coating electrolyte interface) can effectively reduce polysulfide release in ether electrolytes, which is a critical factor in mitigating interface-related issues. The use of conformal polycarbonate-CEI derived from cyclic carbonate can significantly reduce the fatal shuttle effect. This safeguarding mechanism ensures the solid-phase mechanism of SPAN [129] (Fig. 7d). Inspired by this, a tailored electrolyte also promotes the formation of a bilayer SEI with improved Li^+^ ions transport and mechanical strength. This mechanism enables the compatibility of an ultra-thin Li anode [130], achieving high capacity SPAN cathodes (4.08 mAh cm^−2^).

Therefore, manipulating the interfacial chemistry of SEI by optimizing the electrolyte and designing the cathode rationally can develop high-performance, high-load Li–S batteries. Indeed, the SEI layer formed acts as a physical barrier, isolating sulfur species and carbonate solvent. This leads to the desolvation of Li^+^ and prevents solvent molecules from attacking S. Consequently, it prompts the solid-phase lithiation process. Conversely, in the absence of SEI formation, solvated Li^+^ will readily enter the S-active substance, causing LPS formation and severe shuttle effect. For instance, Se doping S_22.2_Se/Ketjenblack (KB) cathodes in HFE-based electrolyte (1,1,2,2-tetrafluoroethyl-2,2,3,3-tetrafluoropropyl ether replaced DME) formed robust SEI on the KB surface, which avoids active materials and the electrolytes contact, inducing the solid–solid lithiation process [131]. However, DME-based electrolytes do not form an SEI, which leads to the formation of LPS and a lithiation process of solid–liquid-solid in Fig. 7c. Thus, the cell composed of S_22.2_Se/KB and HFE-based electrolyte have the potential to achieve a higher reversible capacity in long-term and high-rate cycling owing to minimal shuttle effects. In Fig. 7e, f, spatially confined selenium–sulfur cathodes were lithiated conversion from solid–liquid-solid to solid–solid, as confirmed by in situ characterization techniques. Under this mechanism, SEI membranes can be extended to large mesoporous or even macroporous materials that have been deemed unsuitable for sulfur host, which broadens the way for achieving high volumetric energy density batteries.

To mitigate the shuttle effect of LPS, separators function similarly to fences, impeding the transfer of LPS. In this regard, the long-chain LPS are either fixed by physical barrier and chemical adsorption on the coating material of the modified layer or pushed by electrostatic repulsion toward the cathode side.

#### Implementing Electric Repulsion Strategies for LPS Confinement

**Fig. 8:**
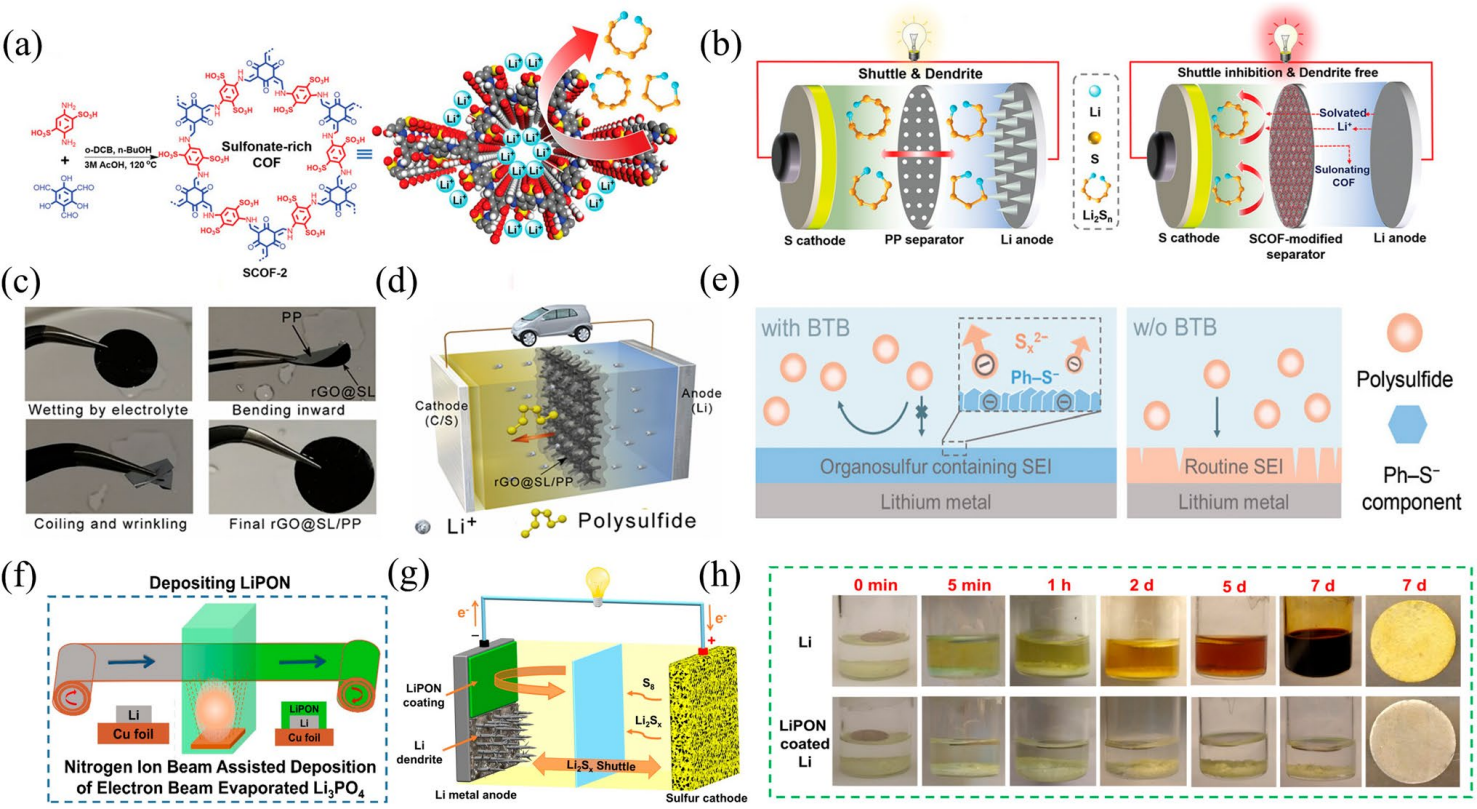
**a** Preparation process of the sulfonated COFs; **b** Graphic comparison of the batteries with different separators [132]; Copyright 2021, Wiley–VCH. **c** Flexibility test and **d** Schematic diagram for inhibiting LPS shuttling effect of the rGO@SL/PP [133]. Copyright 2018, Cell Press. **e** Effect of SEI in shielding LPS of lithium anodes with and without BTB additive [134]. Copyright 2020, Wiley–VCH. **f**, **g** Illustration of the role and coating process of LiPON-coated Li metal anode; **h** Optical images of Li metal foil coated and uncoated with LiPON after soaking in 1 M sulfur/DME solution for 7 days [135]. Copyright 2019, Elsevier

Two molecules with the same type of charge will repel each other electrostatically. In concrete terms, decorating the separator with negatively charged ions or groups can prevent negatively charged S_*x*_^2−^ ions of LPS from crossing the separator due to repulsive forces, while still not affecting Li^+^ transport. Recently, a sulfonate-rich covalent-organic framework (COF) (SCOF-2) has been used to modify the separator, in which both the soluble polysulfide (polysulfide molecules and polysulfide anions) and the designed SCOF-2 possess strong electronegative properties, repelling polysulfide anions through electrostatic interaction and absorbing polysulfide molecules at the same time (Fig. 8a) [132]. Additionally, SCOF-2 has large layer spacing, which promoted the migration of lithium ions and slowed down the formation of lithium dendrites (Fig. 8b). Similarly, a multifunctional graphene–sodium lignosulfonate (SL) composite membrane (rGO@SL/PP) with a large number of negatively charged sulfonic groups has been designed for inhibiting LPS shuttling and achieving uniform transport of lithium ions (Fig. 8c) [133]. The rGO@SL/PP hindered the transfer of electronegative polysulfide ions by charge interaction without affecting the transport of Li^+^. Thus, the charge repulsion effect of rGO@SL/PP with abundant sulfonate groups strongly suppresses the LPS shuttle, while maintaining uniform transport of Li-ions, which has resulted in highly robust Li–S batteries that exhibit stable cycling performance over 1000 times, even at a high current density of 5 mA cm^–2^ (Fig. 8d).

### Preventing LPS from Contacting the Anode

Avoiding contact of polysulfides with the anode would be a last resort strategy if the shuttling effect cannot be completely prevented by the aforementioned strategies. The shuttled long-chain polysulfide will directly react with lithium metal to form low-order polysulfide, which results in the reduction in active material and deterioration of capacity. Furthermore, the by-products generated by the reaction of polysulfide and lithium are deposited continuously on the surface of the lithium metal anode, which increases the impedance of the Li anode/electrolyte interface and reduces the interface ion transport efficiency. The side reactions among large households, LPS and electrolytes will also produce gas by-products, mainly H_2_ and CH_4_, which are easily confined in the porous lithium deposits, resulting in the increased internal pressure of lithium metal. Thus, constructing the last line of defense (SEI layer) on the lithium metal anode side is an effective method for avoiding reaction of polysulfide in direct contact with lithium metal and deposition of product Li_2_S_2_/Li_2_S on the surface of lithium metal.

#### Construction of SEI Films for Effective Anode Protection and LPS Prevention

Tailoring liquid electrolytes to construct a thin, strong and stable SEI film on the surface of the lithium anode is an efficient measure to avoid corrosion of lithium anode by LPS, and thus inhibit the side reaction. For instance, an organosulfur-containing SEI was tailored by employing 3,5-bis(trifluoromethyl)thiophenol (BTB) additive for shielding of Li metal from the soluble LPS corrosion [134]. In Fig. 8e, the lithium metal undergoes a reaction with the active sulfhydryl group present in the BTB additive resulting in the formation of a Ph–S component, which forms an electrostatic repulsion with the polysulfide anion. As a result, the organosulfur-containing SEI can decrease the depletion of fresh lithium and electrolyte by avoiding side reactions between Li metal and LPS. So as to deliver an initial areal capacity of 4.0 mAh cm^–2^ (950 mAh g^–1^) and keep 3.0 mAh cm^–2^ (700 mAh g^–1^) after 82 cycles at 0.1 C.

In addition, another successful way involves coating Li metal anode with carbon-based interlayers [136], creating a solid electrolyte protection layer [137] and adjusting the composition of the electrolyte to prevent the dissolution of LPS. The protective layer consisting of a dense and average lithium phosphorus oxynitride (LiPON) has been prepared by nitrogen plasma-assisted electron beam evaporative deposition method (Fig. 8f) [135]. As a protective layer with high ionic conductivity, chemical stability and mechanical strength, LiPON can effectively prevent corrosion reactions between lithium metal and organic electrolytes. It also promotes the average deposition or dissolution of lithium metal (Fig. 8g, h). This results in a stable cycle life of lithium metal symmetrical battery at a current density of 3 mA cm^–2^ for over 900 cycles without any lithium metal dendrite formation. Moreover, the LiPON-coated lithium metal as the anode can also be used to prepare~300 Wh kg^–1^ high-performance Li–S pouch cell.

## Concrete Strategies to Inhibit the Shuttle Effect in Li–S Batteries

The infamous shuttle effect and slow kinetics have long hindered the practical application of Li–S batteries. Since Li–S batteries are secondary batteries with multi-step reactions, the shuttle effect and slow kinetics affect all parts of the battery components. Researchers have applied various strategies to different components of the battery including designing carbon matrices at the nanoscale [28, 84, 138], using metal oxides [139–141]/chalcogenides [141–143]/nitride as interlayers or hosts, among others. And the following strategies are categorized according to Sect. 3 to avoid the shuttle effect at different cell components for Li–S batteries.

### Rational Construction of Sulfur Cathodes

Sulfur cathode is a vital element in Li–S batteries for it performs a key function by releasing capacity, increasing energy density, and improving cycle life. Prevention of diffusion of soluble polysulfides is the primary approach for suppressing the shuttle effect. Various sulfur cathode materials with specific properties have been designed to inhibit the shuttle of LPS. As described in Sect. 3, sulfur cathodes can inhibit the shuttle effect by spatially confining and chemically anchoring LPS, using catalysts to accelerate the reaction kinetics for effectively enhancing battery performance.

#### Short-chain Sulfur Cathodes

**Fig. 9:**
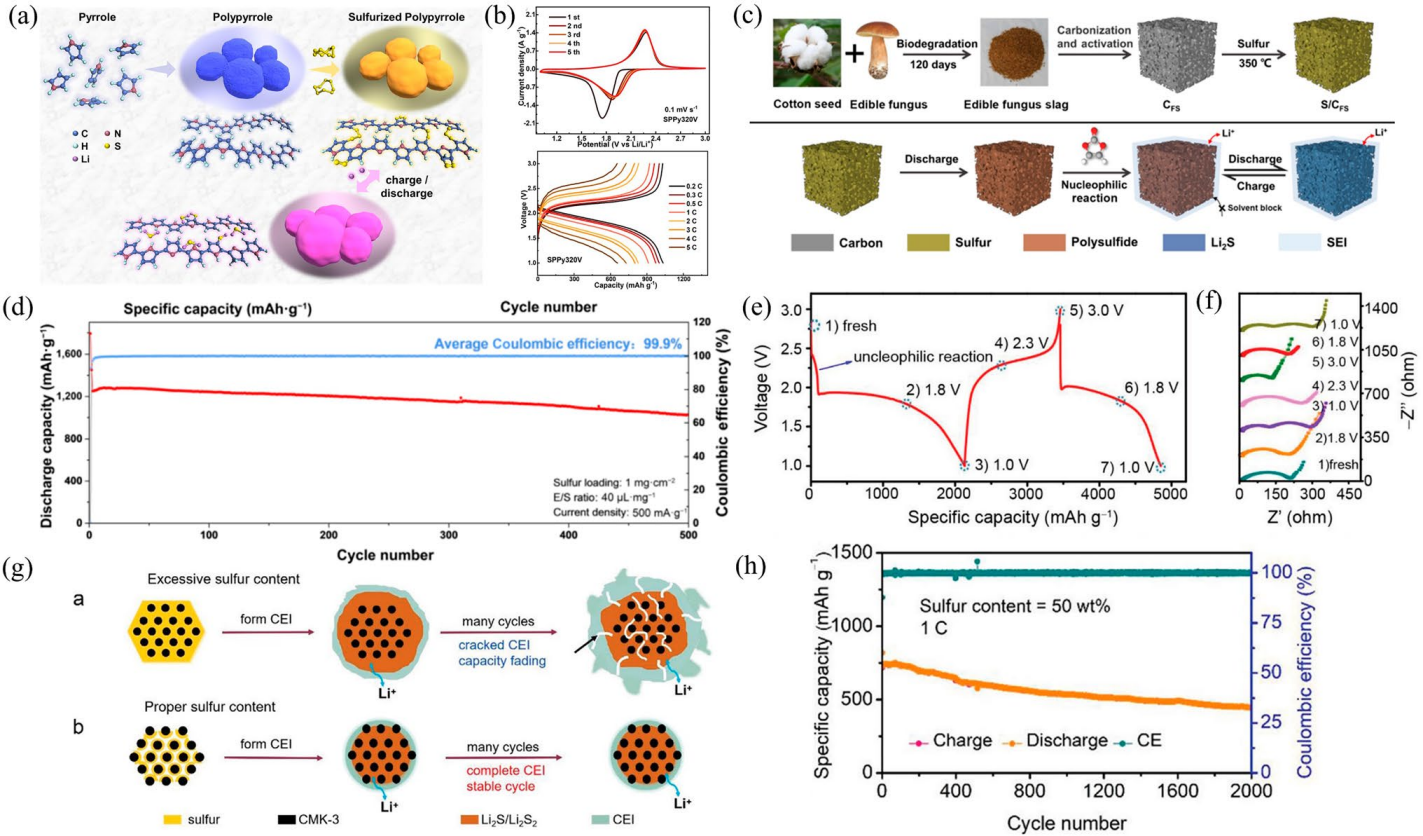
**a** Preparation process of sulfurized polypyrrole. **b** CV curves and discharging/charging curves of rate performance the SPPy320V cathode [144]. Copyright 2023, Elsevier. **c** Synthetic process and the reaction mechanism of the S/C_FS_ cathode in the VC-ether co-solvent electrolyte **d** The electrochemical performance of the S/C_FS_ cathode [145]. Copyright 2023, Spring Link. **e** The plateau curves and **f** Nyquist plot of the impedance spectra (sulfur content = 70 wt%). **g** Schematic illustrations of CEI with 70 wt% and 50 wt% sulfur content. **h** The electrochemical performance with appropriate sulfur content [146]. Copyright 2022, Wiley–VCH

To prevent the initiation of the shuttle effect, which involves the production and degradation of LPS, one possible strategy is to avoid the formation of soluble long-chain polysulfides in the cathode. This can be achieved by physical confinement of small sulfur molecules (S_2−4_) in micro-compartments or by chemical attachment of short-chain sulfur species to the polymer backbone through covalent bonding. Short-chain sulfur polymers can be formed when there are unsaturated bonds or dehydrogenating with sulfur to release hydrogen sulfide (H_2_S) during the pyrolysis, which is similar to other organosulfur compounds like SPAN, SPANI and sulfurized aminophenol–formaldehyde resin (SAF). However, the low conductivity and ionic conductivity of organosulfur compounds impede the kinetics of the SRR, leading to poor rate performance. Novel sulfated polypyrrole (SPPy) compounds were introduced based on pyrolysis and dehydrogenation behaviors [80], in which short-chain sulfur was successfully added to the backbone of SPPy distinct from the pristine S_8_ form in conventional sulfided polypyrrole (PPy@S) blends (Fig. 9a). The material has both a solid–solid transition mechanism and superior lithium ion and charge transfer kinetics [144]. Therefore, the samples obtained at 320 °C (SPPy320V) exhibited initial capacity of 803 mAh g^−1^ at a high rate of 2 C, and the decay rate was 0.022% per cycle during 700 cycles (Fig. 9b).

#### Interfacial Interaction in Sulfur Cathode

Another strategy is to use the electrode design in conjunction with electrolyte modulation for forming a dense SEI film on the surface of cathode by appropriate nucleophilic reaction of LPS with the electrolyte at the early stage of discharge. This research suggests that using edible fungal sludge-derived porous carbon (C_FS_), paired with vinyl carbonate (VC) as a co-solvent for the ether-based electrolyte, could be an effective strategy in producing a protective layer onto the surface of the S/C_FS_ composites in situ. This protective layer could isolate the internal sulfur from the external electrolyte, inhibiting any further generation of soluble LPS (Fig. 9c). This enables the system to function in a solid–solid conversion mode, resulting in a high reversible capacity of 1557 mAh g^-1^ along with 99.9% high cycling efficiency over 500 cycles (Fig. 9d) [145].

The CEI produced onto the sulfur cathode surface plays a prominent role in the solid-phase conversion in Li–S batteries, which can effectively prevent the dissolution of LPS. Figure 9e shows the charging-discharging curve for a cathode with 70 wt% sulfur. During the first discharge, the newly generated LPS underwent a 2.25 V nucleophilic reaction with the limited carbonate solvent, and the reaction products participated in the formation of CEI (Fig. 9e). The corresponding electrochemical impedance spectroscopy (EIS) curve shows that CEI is present in subsequent operations of the battery (Fig. 9f). However, excess sulfur (70 wt%) can cause the formed CEI to crack due to large volume changes caused by repeated reactions during cycling (Fig. 9g-a), which triggers continued decomposition of the electrolyte and nucleophilic reactions between the LPS and the carbonate solvent [147, 148]. This will result in the formation of thick CEI, thus reducing the cycle life. In contrast, the volume of the reduction product (Li_2_S) under the appropriate sulfur content does not exceed the host volume (Fig. 9g-b), and the biphasic conversion reaction between solid phases based on the CEI strategy has the advantage of prolonging the cell life [146]. Thus, if the sulfur content is sufficient (50 wt%), the assembled battery (the sulfur host used was CMK-3, which has a single pore-size distribution centered among 2.5–4.3 nm) achieves an initial capacity of 819 at 1 C and after 2000 cycles maintains a capacity of 445 with a attenuation rate of only 0.03% (Fig. 9h). Moreover, this strategy for CEI demonstrates that the battery could operate at lean E/S conditions. At lean electrolyte (E/S = 3 µL mg^−1^) and sulfur content of 60 wt%, the cell provided a high initial areal capacity of 7.4 mAh cm^−2^ under sulfur loading of 4.3 mg cm^−2^.

#### Pysical-chemical Confinement of Sulfur Cathode

**Fig. 10:**
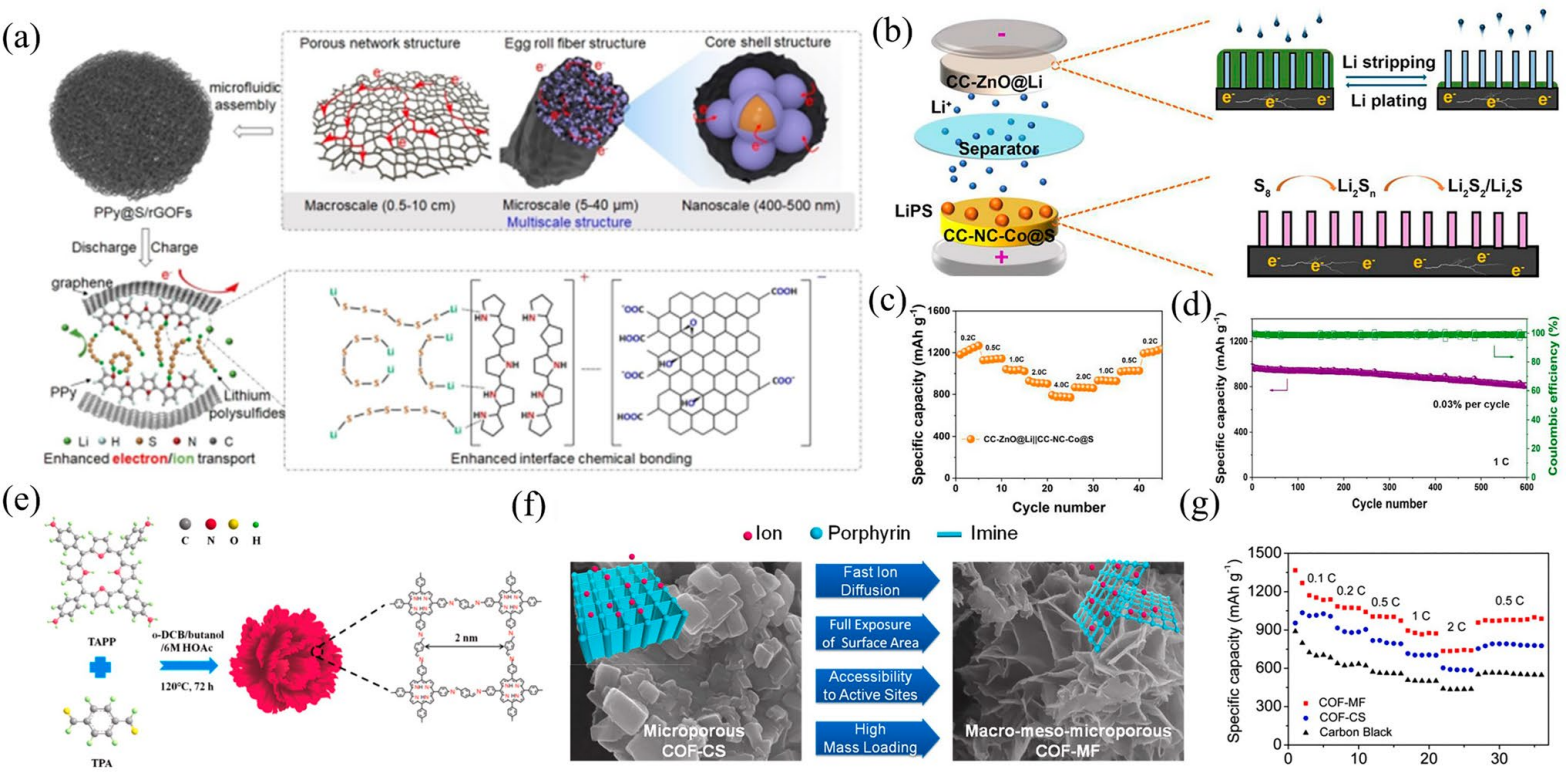
**a** Interface illustration of PPy@S/rGOFs [149]. Copyright 2022, Wiley–VCH. **b** Schematic illustration and **c**, **d** electrochemical performance of the CC-ZnO@Li||CC-NC-Co@S full battery [150]. Copyright 2023, American Chemical Society. **e** Schematic illustration of the preparation of COF-MF; **f** Structural features and **g** rate capability of COF-MF and COF-CS [151]. Copyright 2019, Elsevier

The integration of multiple multidimensional nanostructured materials as excellent hosts for sulfur is a promising strategy. Recently, a synergistic interface bonding enhancement strategy has been enabled by designing a novel sulfur cathode has been developed in a flexible fiber-shape composite form, where using a simple microfluidic assembly technique, uniformly distributed mono-disperse nanospheres (~ 500 nm) of polypyrrole@sulfur (PPy@S) were implanted into the internal cavities of self-assembled reduced graphene oxide fibers (rGOFs). (Fig. 10a) [149]. Notably, in this flexible core–shell structure, both sulfur nanospheres and LPS are confined to the carbon interface (rGOFs) and the polymer interface (PPy) because of the enhanced interfacial chemical bonding which endows the excellent adsorption ability. Interestingly, by wrapping a controllably prepared GO sheet around the outer layer of hollow mesoporous spheres with sulfur (HMCS/S), the advantages of their respective structures can be integrated. The HMCS/S@GO electrode exhibited an initial discharge capacity of 1054 mAh g^−1^ at 0.5 C, its capacity retention of 60.2% after 100 cycles is higher than that of the HMCS/S electrode which is 54.7%. The GO layer acts as an additional physical barrier and chemical trap for polysulfide intermediates, which in turn reduces charge/discharge shuttling and improves conversion kinetics [100]. Novel multifunctional LSB cathode hosts were used, which utilized bronze TiO_2_ nanosheets (TiO_2_–B) to firmly anchor LPS and promote its rapid redox transformation. TiO_2_-B has a strong chemical affinity for polysulfides because of its exposed (100) surfaces and Ti^3+^ ions, which effectively restrict LPS to its surface. The combined cathode has better electronic conductivity. This is due to Ti^3+^ ions and interfacial coupling with carbon, which enhance redox conversion kinetics. Thus, the TiO_2_–B/S cathode showed a high capacity of 1165 mAh g^−1^ at 0.2 C, outstanding rate efficiency of 244 mAh g^−1^ at 5 C [98].

#### Heterojunction Sulfur Cathode

The current mainstream strategy for addressing cathode challenges involves the development of multifunctional cathode hosts utilizing physical confinement, chemical anchoring, and prominent electrocatalytic properties. On carbon cloth (CC), two MOFs based on Zn and Co were synthesized: CC–Co–ZIF–L (as a host for S with Co nanoparticles incorporated within the carbon backbone (CC–NC–Co)) and CC–Zn–ZIF–L (as host for lithium metal with lithiophilic ZnO arrays (CC–ZnO)), respectively (Fig. 10b) [150]. On the cathode side, the presence of C nanosheet skeleton enables the confinement of LPS and the enhanced polarization owing to Co nanoparticles embedding further accelerates the redox kinetics of LPS. Thus, in Fig. 10c, d, Li–S half-batteries with CC–NC–Co@S cathodes delivered outstanding rate capability (746 mAh g^−1^ at 4 C) and long-term stable circulation (capacity retention of 90.8% after 500 cycles). Moreover, the full cells with CC–NC–Co@S cathode and CC–ZnO@Li anode have exhibited exceptional rate capability (793 mAh g^−1^ at 4 C) and impressive long-term stabilities (per-cycle capacity degradation 0.02% when at 0.5 C cycling 900 times).

#### Polymer-based Sulfur Cathode

Covalent organic frameworks (COFs) are commonly used as hosts with sulfur-redox confinement to enable varied states during cycling of highly efficient Li–S batteries. 3D hierarchical flower superstructures (COF-MF) containing porphyrin-rich conjugated ultra-thin nanosheets were firstly bottom-up synthesized as a multi-scale engineering solution to fully demonstrate the potential of COF in Li–S batteries (Fig. 10e) [151]. With minimal nanosheet stacking, unique macro–meso–micro porosity, and large accessible specific surface area, COF-MF not only transforms COF from conventional diffusion-dominated redox kinetics to a charge transfer-controlled process, but also fully exposed porphyrin then serves as a unique anchoring site to maximize the chemisorption of polysulfides and improve sulfur utilization (Fig. 10f). Therefore, the COF-MF, as a polymer host, endowed Li–S batteries excellent ultra-stable cycles (0.047% ultralow decay rate over 1000 cycles at 1 C) and appealing areal capacity (4.78 mAh cm^−2^ at a sulfur loading of 4.1 mg cm^−2^), much superior to the bulk COF counterpart (attenuation of 0.13% over 400 cycles at 1 C) (Fig. 10g).

#### Catalyst-enhanced Sulfur Cathode

**Fig. 11:**
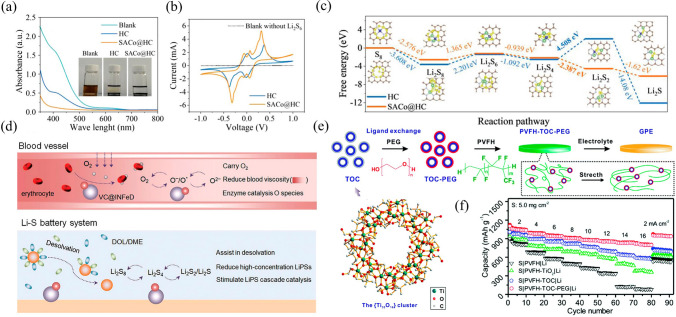
**a** UV–VIS spectra of Li_2_S_6_ solutions after interactions with HC and SACo@HC; **b** CV curves of the symmetric cells in electrolyte with 0.5 mol L^−1^ Li_2_S_6_ and without Li_2_S_6_ at a scanning rate of 1 mV s^−1^; **c** Free energy of LPS on HC and SACo@HC substrates [153]. Copyright 2022, Wiley–VCH. **d** Schematic illustration of the design inspiration of VC@INFeD molecular catalyst [156]. Copyright 2022, Wiley–VCH. **e** Synthesis and **f** rate capabilities in PVFH-TOC-PEG electrolyte [157]. Copyright 2021, The Royal Society of Chemistry

Furthermore, single-atom catalysts (SAC) offer significant potential for catalyzing the polysulfide conversion reaction kinetic owing to their maximum atom utilization efficiency (≈100%) and unique catalytic properties [152]. A sulfur host has been developed in the form of a cobalt single-atom catalyst supported on heteroatom (O, N, S) codoped carbon (SACo@HC) with unique CoN_3_S-active moiety [153]. The SACo@HC is comprised of sulfiphilic and numerous lithiophilic active sites that form Li–O, Li–N, Li–S, Co–S bonds, which can efficiently facilitate the adsorption of LPS (Fig. 11a). As shown in Fig. 11b, in the cyclic voltammograms (CV), symmetrical cells with HC and SACo@HC exhibit eight redox peaks representing four steps during LPS conversion process (S_8_ ↔ Li_2_S_8_ ↔ Li_2_S_6_ ↔ Li_2_S_4_ ↔ Li_2_S) [154]. However, the SACo@HC showed higher peak current density, indicating that during the process of solid–liquid transform, SACo@HC displayed greater catalytic activity. As displayed in Fig. 11c, the SACo@HC exhibited lower reduction potentials for LPS compared to HC, demonstrating that atomically dispersed cobalt centers (CoN_3_S) can encourage the conversion of LPS with faster kinetics and lower polarization. A high capacity of 1425.1 mAh g^−1^ at 0.05 C and an excellent rate performance of 745.9 mAh g^−1^ at 4 C were obtained for the S-SACo@HC composite with 80 wt% sulfur loading. In addition, Ni single atoms supported on N-rich mesoporous carbon (Ni-NC(p)) can also act as sulfur host for Li–S batteries [155]. The unique architecture design, N-atom doping and Ni single-atom catalyst synergistically achieved physical confinement, chemical adsorption and catalytic transformation, which suppressed the shuttle effect and accelerated the redox kinetics of LPS. Therefore, the Ni–NC(p)/S delivered an average discharge capacity of 778.1 mAh g^−1^ at 1 C.

#### Desolvated Sulfur Cathode

Although various catalytic materials (e.g., heterojunctions, heteroatom (N, O, S)-doped carbon, and single atoms catalysts (SACs)) have been simply combined to overcome their respective weaknesses in stepwise SRR, the lack of interfacial connectivity and charge transfer between compounds are still the limiting factors in improving the kinetics of electrode reactions [77, 158]. Thus, designing an electronic reservoir that can release and accept electrons from sulfur species during discharging and charging can efficiently activate stepwise and reduce the activation energy, thus providing an ideal solution for smooth and sustainable catalyzing SRR for Li–S batteries [159]. As a result, a highly efficient VC@INFeD catalyst incorporated on the sulfur cathode with assistance of multiple H/Li-bonds has been developed at the cathode/electrolyte interface. VC@INFeD is capable of capturing dissolved LPS clusters present at the cathode/electrolyte interface through H-bonds, resulting in a local high-concentration distribution. With the assistance of Li-bonds and Fe^2+^/Fe^3+^ activity centers, the captured LPS clusters are rapidly transferred and efficiently converted at the gradient catalytic site. In particular, the two components, INFeD and VC, are catalytic for long-chain and short-chain polysulfides, respectively (Fig. 11d). Therefore, even when subjected to lean electrolyte (approximately 7 µL mg^−1^) and high-sulfur loading (5.2 mg cm^−2^), VC@INFeD significantly reduced energy barrier for each step of the redox process, suppressing the shuttle effect, and imparting a high utilization of sulfur and excellent cycling stability (441 mAh g^−1^) in Li–S cells [156].

### Tailoring Electrolyte Systems

Modulating the electrolyte suppresses both shuttle effect and the formation of lithium dendrites for Li–S batteries. Electrolytes play a key role in determining cathode and anode chemistry [160, 161]. Researchers have proven that SEIs formed on both sulfur cathodes and lithium anodes are inadequate for long-term cycling using conventional, organic electrolytes. Electrolyte modulation has become prevalent in the literature for adjusting the surface chemistry of active materials and enabling reversible reaction sites.

#### Co-solvents Electrolyte Systems

In Li–S batteries, the nature and quantity of solvents in the electrolyte play a vital role because they function as the medium for Li^+^ transport and are extensively involved in lithium salt reactions on electrode surfaces. A well-designed electrolyte should suppress shuttle of LPS and safeguard Li anode, thereby extending the cycling duration of Li–S batteries [162]. Long-chain LPS can be dissolved in conventional ether-based electrolytes and move to the anode, whereby they react with the lithium metal, leading to a decrease in capacity and an increase in resistance. Recently, high concentration electrolytes (HCEs) with dilute solvents have been developed to inhibit the dissociation of LPS [161, 163]. Nevertheless, the extensive use of costly lithium salts in HCE electrolytes has given rise by several challenges such as high cost, low ionic conductivity, poor wettability and high viscosity of LPS [58, 164]. In order to solve these problems while maintaining limited solubility of LPS, the proposal suggests localized high-concentration electrolytes (LHCEs) by supplementing fluorinated solvents having weak donating ability (e.g., fluorinated ether and fluoride benzene) to HCEs. As an illustration, fluorinated ethers (1,1,2,2-tetrafluoroethyl-2,2,3,3-tetrafluoropropyl ether, TTE) have been used as co-solvents in electrolytes, where LPS are trapped within the cathode due to the formation of fluorine-rich SEIs (e.g., LiF), which improved the cycling performance and suppressed the LPS shuttle [165]. Additionally, TTE was also used to dilute a dual-salts based medium-concentrated electrolyte (MCE) to form diluted medium-concentrated electrolytes (DMCEs). Thanks to the LiF-rich SEI film formed by TTE and the effect of inhibiting side reactions, LSB batteries delivered an initial capacity of 682 mAh g^−1^ in the DMCE electrolyte (667 mAh g^−1^ in MCE) with 92% capacity retention and lifespan of 500 cycles [166].

#### Quasi-solid Electrolytes

Gel polymer electrolytes (GPEs) are a type of quasi-solid electrolytes, which are interfacially compatible with both the cathodes and anodes and exhibit enhanced ionic conductivity. They consist of liquid electrolyte enclosed in a polymer matrix [167–170]. The electrolyte solvent in GPEs facilitates solid/liquid interfacial sulfur conversion [61, 135, 171, 172]. While GPEs enable the creation of high-sulfur-loading Li–S batteries, plastification of the liquid electrolyte can significantly decrease the GPEs mechanical stability. To reinforce GPEs, it is common practice to introduce inorganic nanoparticles (e.g., TiO_2_, Al_2_O_3_, and SiO_2_) into the polymer matrix [173, 174]. An example is a GPE reinforced with a titanium-oxo cluster (TOC) that was prepared for constructing low E/S ratio Li–S batteries [157] in Fig. 11e. The designed TOC promotes the behavior of the polymer matrix in forming a film, inhibiting polysulfide shuttling, and leading to uniform Li deposition. In the first cycle at 2 mA cm^−2^, the discharge capacity of the S|PVFH-TOC-PEG|Li cell was as high as 1103 mAh g^−1^ calculated based on the mass of sulfur. Even increasing to16 mA cm^−2^ such high current density, the S|PVFH-TOC-PEG|Li cells maintained 802 mAh g^−1^ such a high specific capacity (Fig. 11f).

#### Dual-phase Electrolyte Approaches

**Fig. 12:**
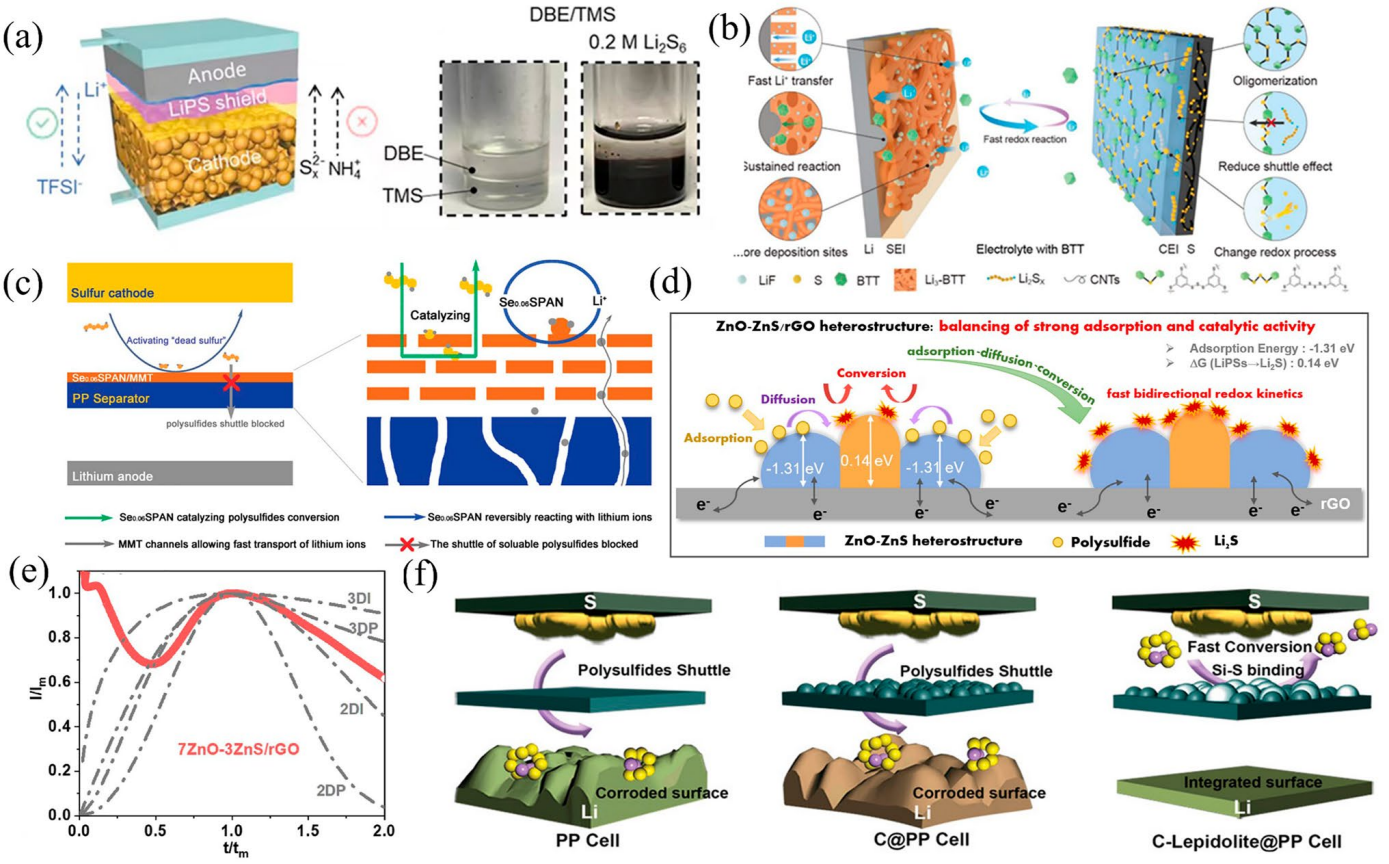
**a** Schematic and photographs for the immiscibility of Li_2_S_6_ in TMS solution and DBE [175]. Copyright 2022, Wiley–VCH. **b** Dual SEIs formed with BTT electrolyte additive [176]. Copyright 2021, Nature. **c** Schematic illustration of the Se_0.06_SPAN/MMT@PP separator for Li–S battery [177]. Copyright 2022, Wiley–VCH. **d** The operating principle in Li–S cells with ZnO–ZnS/rGO heterostructures functionalized separators. **e** Dimensionless transient curves of 7ZnO–3ZnS/rGO with theoretical models [178]. Copyright 2022, Elsevier. **f** Schematics for the absorption and conversion of LPS on PP, C@PP, and C-Lepidolite@PP separators [179]. Copyright 2021, Wiley–VCH

The dissolution of LPS is crucial for rapid cathode kinetics, particularly under lean electrolyte conditions, although it jeopardizes anode stability. According to the phenomenon of phase separation between different polar solvents and the mediator-solvating property, tetramethyl sulfone (TMS) and dibutyl ether (DBE) have been selected as dual-phase electrolyte system for Li–S battery. Specifically, the high-density TMS-LiTFSI with high-polarity acted as the cathode electrolyte, whereas the anode electrolyte consisted of DBE and a polymeric ion conductor in GPE. The cathode electrolyte can strongly solvate LPS and propel SRR process [175]; meanwhile, the corrosive species such as LPS and ammonia trifluoroacetate can be effectively discouraged by the DBE on the anode side, allowing the activity and stability of batteries to be significantly increased (Fig. 12a). Consequently, pouch cells assembled with the dual-phase electrolytes have delivered 120 cycles under a low-Li-excess condition (N/P = 3) and lean electrolyte (4 µL mg^−1^).

#### Electrolyte Additives for Shutle Inhibition

Electrolyte additives that are appropriate should have the ability to carry out electrochemical conversions and produce functional surface films on both electrodes. 1,3,5-benzenetrithiol (BTT) has been employed in the fabrication of dual SEIs (D-SEIs) as electrolyte additives (Fig. 12b), which forms SEI at the anode by reacting with Li metal and meanwhile self-assembling with sulfur at cathode creates an adaptable monolayer on homogeneous surfaces by forming interfacial layers containing S-Li and S–S bonds [176]. These in situ formed bonds changed the redox pathway of the sulfur cathode and highly regulated Li deposition/stripping behavior, thus achieving improved performance. The Mulliken charge distribution of BTT showed that the charge has been transferred from Li electrode to the S counterpart upon adsorption, resulting in stronger ionic bonding. The discharge capacity of Li–S cell with BTT was as high as 1239 mAh g^−1^ and long-term stable cycling was over 300 cycles at 1 C.

### Constructing Functional Separators

Separators play a pivotal role in preventing the dissolution and diffusion of LPS when cathode and electrolyte conditioning strategies are insufficient. Nevertheless, commercial separators, like the polypropylene porous membranes (PP) or polyethylene porous membranes (PE), have pore sizes as wide as 100 nm, which is much larger than that of the long-chain LPS with an average size of several nanometers, resulting in the easy penetration of LPS to the anode side. Therefore, almost any typical host material is possible to be employed to optimize a separator or architecture an interlayer.

#### Multifunctional Materials Coating for Separators

Depositing functional materials on the separator to trap soluble intermediates is a common strategy to suppress LPS. However, due to the transformation barrier of insoluble Li_2_S and Li_2_S_2_, the captured LPS tend to exit in the subsequent electrochemical process and accumulate as “dead sulfur” on the separator over time [180, 181]. Furthermore, the accumulated “dead sulfur” significantly obstructs the separator's lithium-ion transport channels, leading to clear degeneration in the performance [182–186]. Some catalytically active modifiers have been discovered to alleviate the “dead sulfur” issue via accelerating the conversion of LPS [187–189]. The optimizing modification separators with multifunctional coating layer composed of bicomponent composite comprising montmorillonite (MMT) and selenium-doped sulfurized polyacrylonitrile (Se_0.06_SPAN) efficiently enabled the catalytic activation of the blocked LPS and prevented the cumulative of “dead sulfur” [177]. The layered structure of MMT serves as an effective anchor for dissolved polysulfides while allowing the migration of lithium ions (Fig. 12c). It also served as a support to minimize volume changes as Se_0.06_SPAN charged and discharged. Moreover, MMT-loaded Se_0.06_SPAN accelerated the conversion of anchored polysulfides and activated “dead sulfur” owing to the potential barrier of insoluble Li_2_S and soluble LPS was reduced. As a result, the Li–S battery with the Se_0.06_SPAN/MMT@PP delivered a low fading rate of 0.034% during 1000 cycles. Furthermore, under high-sulfur loading (26.75 mg cm^−2^) and lean electrolyte (4.5 µL mg^−1^), the battery achieved a super-high areal capacity of 33.07 mAh cm^−2^.

In order to diminishing the shuttling effects in Li–S batteries, it has been found helpful to add catalysts to the separator to accelerate the conversion of LPS. However, it is challenging to achieve both high catalytic activity and strong adsorption using a single catalyst. The modified heterostructure of ZnO-ZnS/reduced graphene oxide (rGO) with strong balanced adsorption and high catalytic activity has achieved significantly enhanced polysulfide conversion and Li–S performance (Fig. 12d) [178]. In addition, through a controlled vulcanization, the ZnO-ZnS/rGO heterostructure achieved the optimal overall performance with a ZnO/ZnS ratio of 7:3, suggesting a balance between adsorption and catalytic activity. ZnO-ZnS/rGO heterostructures can significantly improve redox kinetics and inhibit polysulfide shuttling when used as functional coatings on separators. As shown in Fig. 12e, under the synergistic effect of the heterostructure, the Li_2_S deposition pattern showed a mixed model of 3DP and 2DI [9, 190], demonstrating that the heterostructure realized a bidirectional redox reaction between Li_2_S and LPS. Thus, the pure sulfur cathode matched that the 7ZnO-3ZnS/rGO-modified separator delivered initial specific capacity as high as 1186 mAh g^−1^ at 0.5 C and possessed 0.06% fading per cycle over 500 cycles at 1 C.

Forming covalent bonding between S and the atoms from the separators is also an efficient strategy to prohibit the shutting effect. For instance, the designed lepidolite-modified polypropylene (C-Lepidolite@PP) separator can induce the electron transfer from S atoms to the 3p antibonding orbitals of Si atoms, which weakened the S–S bonds and formed strong Si-S bonds, thus effectively confining polysulfides (Fig. 12f) [179]. The lithium-ion diffusion barrier in lepidolite is extremely low (0.081 eV), it permits free migration of lithium ions and this, which in turn improves the conversion of polysulfide from liquid Li_2_S_8_ to solid Li_2_S and fast LPS redox for high-rate current operation. Therefore, Li–S batteries with the C-Lepidolite@PP delivered an excellent areal capacity of 7.53 mAh cm^−2^ under 6.5 mg cm^−2^ sulfur loading and a superior rate performance of 703 mAh g^−1^ at 7 C.

### Modified Lithium Metal Anode

If the approach to eliminate LPS shuttling by the use of optimized sulfur cathode, functional separators, and customized electrolyte compositions is still inadequate, the residue long-chain soluble LPS will inevitably diffuse to Li anode side through a concentration gradient. Once these soluble LPS diffused to the anode surface and reacted with Li metal, it will result in the formation of the insoluble and inert Li_2_S and Li_2_S_2_, resulting in loss of active material, irreversible depletion of Li and reduction in coulombic efficiency. Therefore, introduction of a passivation layer at the interface to inhibit the diffusion of LPS, facilitated fast transport for Li^+^ ions, and prevent the formation of irregular Li dendrites is effective in inhibiting active S species loss and prolong the cycle life of Li–S batteries. It has been described above that the SEI layer is formed by tailoring the electrolyte to prevent the direct reaction of the polysulfide with the lithium metal. This section will show artificial protective layers and other strategies to avoid shuttle effects on negative electrode materials.

#### Artificial Solid Electrolyte Interphase (SEI)

**Fig. 13:**
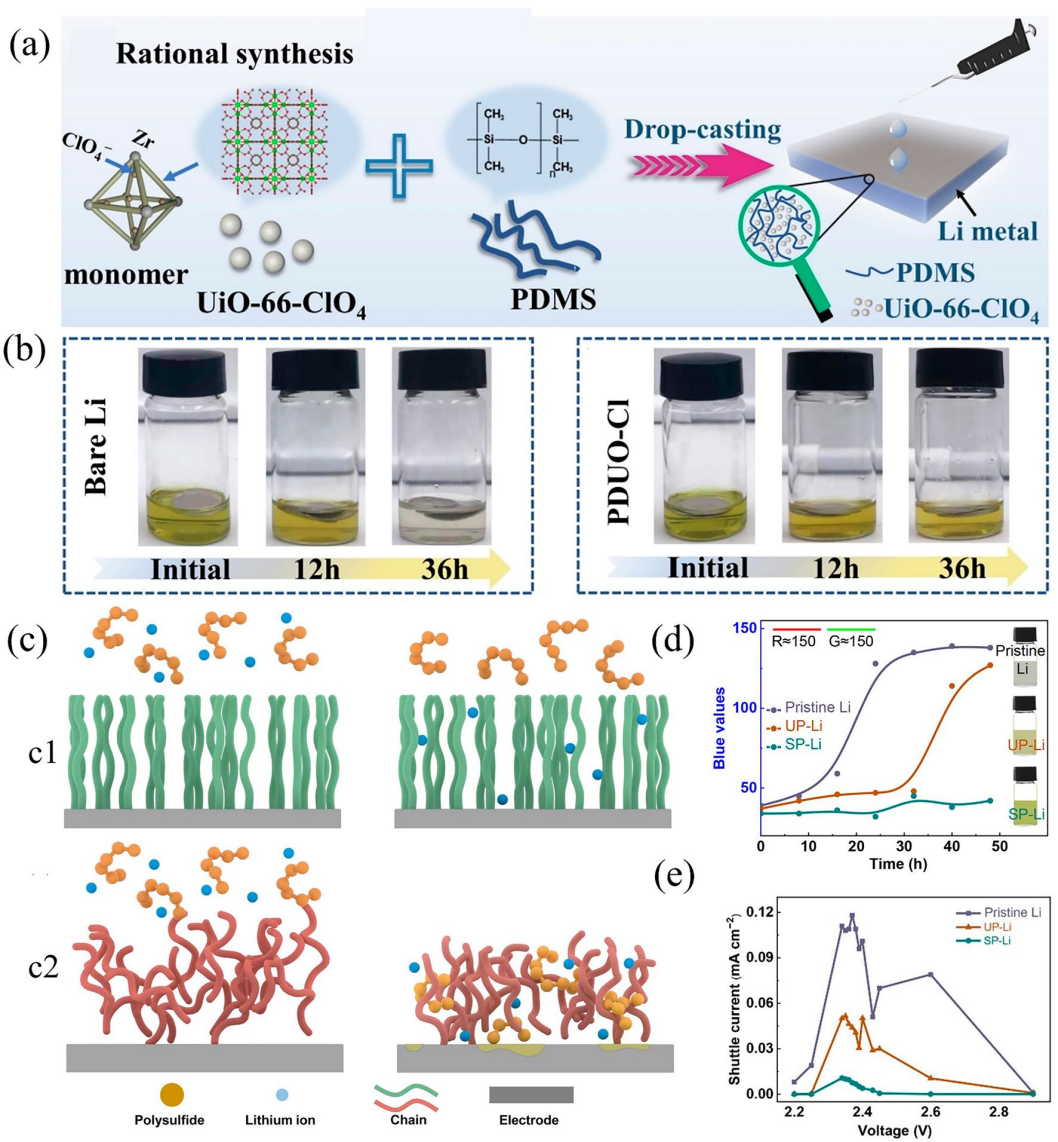
**a** The manufacturing steps of PDUO-Cl protective layer for Li anode. **b** the visualized test of Li_2_S_6_ electrolyte for bare Li and PDUO-Cl@Li [193]. Copyright 2022, Elsevier. **c** The LPS in c1) ordered selective permeable polymer interphase and c2) disordered polymer interphase. **d** The visualized test of Li_2_S_6_ electrolyte with pristine Li, unselective-permeable Li, and selective permeable Li. **e** The shuttle currents in LiNO_3_-free ether electrolyte [194]. Copyright 2021, Wiley–VCH

Numerous organic and inorganic materials have been utilized to fabricate artificial SEIs for the purpose of enhancing the performance of lithium anodes [191, 192]. For example, a stable UiO-66-ClO_4_/PDMS (PDUO-Cl) biomimetic protective layer has been designed to modify Li anode by a drip coating method (Fig. 13a) [193]. When bare Li and PDUO-Cl@Li were immersed into a solution containing Li_2_S_6_, it was observed that the bare Li-immersed solution became almost colorless after 36 h, owing to the reaction of Li metal with LPS. Conversely, the discoloration of the PDUO-Cl@Li-immersed solution was markedly reduced, demonstrating that the LPS in the solution were not exhausted (Fig. 13b). This result suggested that the PDUO-Cl could resist the aggression of LPS. Therefore, the symmetric cells of the PDUO-Cl@Li delivered a stable long-term cycle over than 1400 h at 0.5 mA cm^−2^. The half cells with a PDUO-Cl@Li also showed a relatively high capacity retention of 69% after 100 cycles at 0.1 C.

#### In Situ Ion-selective Interphase Engineering

Except for developing a protective layer for the lithium anode, it is essential to enhance the transportation of Li^+^ ion through the SEI layer as it directly affected the plating/stripping behavior of lithium [181]. In Fig. 13c, parasitic reactions generate disordered Li^+^ channels on the lithium surface, which obstruct ion transportation and promote electrode corrosion [195]. Thus, it is essential to construct ion-selective, ordered channels which permitted Li^+^ to diffuse rapidly across the working interface and prevent large amounts of corrosive anions from passing through [196, 197]. For instance, a direct reaction between aminopropyl-terminated polydimethylsiloxane (AT-PDMS) and lithium metal has been used to create selectively permeable interphase for a lithium anode [194]. The entropic ordered organization of the polymer chain reduces the free volume of the polymer, selectively blocking bigger polysulfides which have stronger spatial barriers, while allowing the passage of Li^+^ ions (Fig. 13d). Without adding a lithium nitrate passivator to the electrolyte, the shuttle current of Li–S batteries reduced by 90% and the coulombic efficiency improved from 82 to 91% (Fig. 13e). Table 2 summarized the recent advancements in Li–S batteries based on the inhibition of the shuttle effect for various components.

**Table 2:** The recent advancements in Li–S batteries based on the inhibition of the shuttle effect for various components of the devices

Cell component	Concrete methods	Materials	Sulfur loading (mg cm^−2^)	Capacity (mAh g^−1^)/C rate/cycles	Fading rate (%)	S loading (wt%)	E/S (μL mg^−1^)	Refs.
Cathode		SPPy320V	1	803/2/700	0.022	39.4	–	[144]
	Short-chain sulfur cathodes	S/C_FS_	1	1027/0.5/500	0.036	80	40	[145]
	Interfacial interaction sulfur cathode	CMK–3/S	4.3			60	3	[146]
		PPy@S/rGOFs	4	865/0.1/200	–	71.3	8	[149]
		3DIO FCSe–QDs@NC	1.5–2	801/1/500	0.035	70.2	20	[88]
	Pysical-chemical confined sulfur cathode	S-TVTCSi_4_-KJB_2%_	1	652/0.5/500	0.037	80.8	25	[89]
		S@CNTS/Ni–PC	2	408/2/500	0.1	30	25	[90]
		Co-NCNT@HC	1.2	750/1.2/1000	0.037	73.5	–	[91]
	Polymer sulfur cathode	sGNC–S	2–3	829.3/0.5/200	0.155	74.3	–	[92]
		Nb_3_VS_6_	1.5	759/2/1000	0.017	–	–	[93]
		S/Ni–Co	3.84	~ 600/1/200	0.082	74.3	8	[94]
	Heterojunction sulfur cathode	NiO–MoS_2_@CNFs	1.5	973.3/1/500	0.018	–	12	[95]
		v-ZnTe/CoTe_2_@NC/S	1.6	742/1/500	0.022	68	20	[96]
		vanadium-doped MoS_2_ (VMS)	7.2	725/1/300	0.06	75	14.7	[97]
	Catalyst sulfur cathode	TiO_2_-B	2	572/0.2/100	0.34	61	10	[98]
		TiO_2_/BaTiO_3_	1.2	541/0.5/500	0.08	58	10	[99]
		HMCS@GO	1	626/0.2/300	0.16	58.9	–	[100]
		SnS_2_@NHCS	1	395/1/500	0.065	69.7	–	[101]
		Fe_3_C/N–CNF@RGO	1.6	781/0.5/300	0.009	62.5	15	[102]
	Desolvated sulfur cathode	VC@INFeD molecular catalyst	4	727/0.05/80	–	–	7	[156]
Separator	Catalyst coating separator	UiO–66D2 modified separators	2	785/1/500	0.03	75	8.9	[198]
		Se_0.06_SPAN/MMT@PP	0.8	782/1/1000	0.034	-	20	[177]
	Heterojunction coating separator	7ZnO_−3_ZnS/rGO-modified separator	1.5	636/1/500	0.06		20	[178]
	Polymer coating separator	C-Lepidolite@PP	1.42	1133/1/450	0.068	56	–	[179]
Electrolyte	Co-solvents electrolyte systems	VC-ether co-solvent	4	1005/1/30	–	80	10	[146]
	Electrolyte additives	Tetrapropylammonium bromide (T_3_Br) electrolyte additive	1.5–2 4.6	590/1/700 855/0.1/100	0.049 0.03	56	20 10	[199]
	Quasi-solid electrolytes	MoS_2_@rGO-GPE	5.2	819/0.1/500	0.04	–	–	[200]
		FDH-based electrolyte	1–1.5	445/1/2000	0.03	50	-	[149]
		PVFH-TOC-PEG	5	802/-/100	–	67	6	[157]
	Dual-phase electrolyte	GPE(DBE) | TMS	4	715/0.4/250	-	–	7.5	[175]
			Current density (mA cm^−2^)	cycle performance (h)	Areal capacity (mAh cm^−2^)			
		PDUO–Cl@Li	0.5	1000	0.5			[193]
Anode	Artificial solid electrolyte interphase	3DIO FCSe–QDs@NC	3	900	3			[88]
		Li@CAJL	1	400	1			[201]
	In situ ion-selective interphase	NiO-MoS_2_@CNFs/Li	5	1000	5			[95]

## Conclusion and Outlooks

If LPS exhibits shuttling behavior in Li–S batteries, it may be challenging to meet practical demands. Consequently, it is urgent and meaningful to gain a more comprehensive understanding of the shuttling process of LPS, which can also be used as guidance for future shuttle effect inhibition design for Li–S battery applications. This review focuses on the shuttle path of LPS and suppressing strategies in Li–S batteries. The designed principles for prohibiting LPS shuttle, including boosting the sulfur conversion rate, confining sulfur or LPS within cathode host, confining LPS in the shield layer, and preventing LPS from contacting the anode have been discussed and summarized. The summarized recent advances of inhibition of shuttle effect in the sulfur host, electrolyte system, separator, and anode protection demonstrated that the designed principles for prohibiting LPS shuttle are highly correlated to the activity and stability for Li–S batteries.

Currently, tremendous advancements have been acquired with respect to Li–S batteries with many breakthroughs for high-energy and long-stability. However, it is still challenging to eliminate all the side reactions, shuttle effects and finally commercialization. Therefore, it is still urgent to develop efficient strategies for Li–S batteries to realize the practical application. Achieving more advanced characterization techniques may be beneficial for exploring of reaction mechanisms, analyzing of interface engineering in-depth and expanding of interdisciplinary research. A more microscopic perspective can provide a complete and thorough comprehension of Li–S batteries, which can be effectively used to eliminate the shuttle effect.

1. Upgrading of characterization technologies. Advanced characterization methods, especially more direct in situ characterization methods, should be developed for Li–S batteries. Currently, traditional EIS and Raman can only understand the macro mechanism of these strategies on polysulfides. More recently, some ex situ characterization techniques have been used to explore the changes in the physicochemical properties before and after the cycles, which help us to get a better understanding of the Li–S battery process. However, the interfacial and structure–activity relationships between polysulfides, catalysts and electrolytes are still unclear. Therefore, more advanced characterization techniques are highly desired, especially the in situ characterization method. For example, the in situ synchrotron radiation technique can be used to investigate the near-free evolution kinetic behavior of monatomic catalysts in electrocatalytic reduction reactions [202], and catalytic sites and reaction processes can be detected through the use of in situ X-ray absorption spectroscopy (XAS) and surface enhanced infrared absorption (SEIRA) spectroelectrochemistry. The electronic state of each metal site can be observed in real time during the voltage change process, which can help to determine the initial reaction conditions and the reaction intermediate process [203].

2. Exploration of reaction mechanism. However, much enhanced performance of Li–S batteries has been achieved through the aforementioned strategies, significantly suppressing the shuttle effect. But awareness of mechanism about inhibition for polysulfide is still backward in terms of theory. In addition, experimental characterization methods can reflect the integrated performance of Li–S batteries, while theoretical simulation can provide profound mechanism insights from the atomic scale, and has become an indispensable tool in the study for Li–S batteries. Density functional theory (DFT) calculations can determine structural stability, calculate the free energy of the reaction, analyze the electronic structure, and simulate the ionization/molecular diffusion or adsorption kinetics [204]. At the same time, high-throughput screening and machine learning have broad application prospects on the research on Li–S batteries, which can inspire and guide the further development in this field [205]. Further exploration of the reaction mechanism will play a guiding role in the material design and theoretical calculation, which can provide a strong basis for cathodes and anodes design strategy, catalyst selection, electrolyte customization, and SEI component construction. At the same time, reaction mechanism will also provide the direction for Li–S batteries to achieve high energy density under high loading and lean electrolyte conditions. This will provide a solid foundation for the commercialization of Li–S batteries in the future.

3. In-depth analysis of interface engineering. Whether it is the three processes of the Li–S reaction: “quasi-solid,” “solid–liquid-solid,” “solid–solid” process, or the construction of CEI on the cathode or SEI film on the anode, it involves the interphase transition process. In-depth analyzing the phase transition of interface engineering is conducive to the targeted design of materials or structures to achieve the theoretical capacity of Li–S batteries. Simultaneously, interface engineering can also improve the shuttle energy barrier of polysulfide, inhibit the loss of active substances, and avoid the growth of lithium dendrites. The morphological changes of the anode surface during operation can be monitored by in situ optical microscope. Based on the finite element method, COMSOL Multiphysics simulation can be carried out to simulate the local current density and the overall electric field distribution to reveal the evolution and failure mechanism of interface engineering [206, 207]. It lays a foundation for the realization of safe and stable Li–S batteries.

4. Expansion of interdisciplinary research. Similar to sulfur cathodes in Li–S battery, many areas of electrochemical energy storage process face challenges in terms of electrodeposition behavior. For example, the alkali metal–chalcogen system, working in a similar way to Li–S battery, namely Li/Na/K/Mg-S/Se/Te, suffers from slow cathode deposition kinetics during discharge [208–211]. In the case of lithium metal anodes, lithium with high rigidity needs to be deposited flat without forming dendrites to prevent battery short circuits [191]. Although the objects of study for metal anodes and sulfur cathodes are usually different, their related strategies, methods, and materials are of high value for achieving ideal electrodeposition. In addition, electrodeposition has long been recognized as an important technology for material synthesis or device manufacturing in many frontier fields such as solar photovoltaic, thermoelectric and sensors [212]. Therefore, implementing interdisciplinary research to inspire more insightful work far beyond the field of energy storage should be very attractive.
